# Impact of News Portrayals of Physicians as Vulnerable on the Public’s Evaluation and Trust in Physicians Under Different Involvement Levels: Quantitative Study

**DOI:** 10.2196/67947

**Published:** 2025-07-09

**Authors:** Qiwei Li, Jie Zhou

**Affiliations:** 1 Institute of Psychology Chinese Academy of Sciences Beijing China; 2 Department of Psychology University of Chinese Academy of Sciences Beijing China

**Keywords:** vulnerable physicians, media portrayals, physician-patient trust, stereotype content model, involvement

## Abstract

**Background:**

News portrayals of physicians, especially in China, often depict them as vulnerable—overworked, with inadequate compensation, or as victims of violence. These portrayals may send mixed signals to the public, yet their impact remains underexplored. Understanding their impact is essential for informing media strategies and improving physician-patient relationships.

**Objective:**

This study investigated how portrayals of physicians as vulnerable influence public evaluations of their competence, warmth, morality, and overall trust and considered the moderating effects of involvement (ie, hospital visit frequency).

**Methods:**

Four studies were conducted. Study 1 (N=492) examined the effects of daily exposure to vulnerable portrayals, and study 2 (N=710) experimentally exposed participants to vulnerable portrayals to directly investigate the causal relationship between exposure and evaluations with involvement as a hypothesized moderator. Study 3 (N=565) manipulated situational involvement using an imagination task, whereas study 4 (N=436) embedded involvement-enhancing content into news articles to improve ecological validity.

**Results:**

Study 1 revealed that among individuals with low or moderate involvement, greater exposure to vulnerable physician portrayals in everyday life predicted more favorable overall evaluations of physicians (low involvement: *B*=0.11 and *P*=.04; moderate involvement: *B*=0.20 and *P*<.001). No significant effect was found among high-involvement individuals (*P*>.68 in all cases), suggesting an inverted U-shaped moderating effect of involvement. Study 2 supported this pattern—vulnerable portrayals had no significant impact among individuals with low or high involvement (*t*_702_<0.49 in all cases; *P*>.15 in all cases) but had marginally positive effects on individuals with moderate involvement (*t*_702_=1.67; *P*=.10; *d*=0.26). Notably, individuals with superhigh involvement (ie, those in hospital settings) evaluated physicians more negatively following vulnerable portrayals (*t*_702_=2.49; *P*=.01; *d*=0.44). Given that nearly 80% of the general population reports low to moderate hospital visits, which is the positive moderating effect range for involvement, studies 3 and 4 targeted this group and tested whether manipulated situational involvement could enhance the effects of vulnerable portrayals. In studies 3a and 3b, participants in the high–situational involvement condition evaluated physicians more positively in the vulnerable portrayal group than in the control group (3a: *t*_401_=2.71, *P*=.007, *d*=0.37; 3b: *t*_154_=3.48, *P*<.001, *d*=0.93), with no effects under low-involvement conditions. Study 4 confirmed that involvement-enhancing vulnerable portrayals elicited more favorable evaluations compared to the control group (*t*_433_=3.14; *P*=.002; *d*=0.37). Across all 4 studies, overall evaluation significantly predicted trust in the medical profession (*B*≥0.38 in all cases; *P*<.001 in all cases), supporting the hypothesized mediation pathway.

**Conclusions:**

The findings reveal a complex relationship between news portrayals of vulnerable physicians and public perceptions moderated by involvement. These results have practical implications for leveraging media to increase public trust and improve physician-patient relationships.

## Introduction

### Background

In today’s multimedia landscape, public perceptions of physicians are significantly shaped by media portrayals, alongside direct personal experiences [1,2]. News coverage in particular frequently presents physicians as vulnerable, especially in the context of health care crises [3]. In China, systemic challenges such as an underdeveloped health care infrastructure and the uneven distribution of medical resources have contributed to a situation in which large hospitals, especially top-tier hospitals, are overwhelmed. Physicians in these institutions often work around the clock, with little rest between consultations and surgeries. As a result, they may not be able to fully attend to each patient’s emotional and informational needs during brief encounters, which can lead to misunderstandings and strained physician-patient relationships. This heavy workload, combined with limited compensation and occasional exposure to workplace violence, imposes significant physical and psychological burdens on physicians.

Vulnerability in this context refers to portrayals of physicians as individuals facing physical, emotional, and systemic challenges such as violent attacks, overwork, and inadequate compensation. These portrayals are increasingly common in Chinese news coverage and are often intended to raise public awareness of the challenges faced by physicians, reduce public hostility, and advocate for health care reform. For example, an analysis of *China Youth Daily*—a national daily newspaper with a youth-oriented focus and wide readership known for its in-depth reporting on social issues such as health care [4]—revealed that, from 2009 to 2018, nearly half of the reports depicted physicians as “misunderstood weak individuals,” “victims of violence,” or as receiving “low remuneration,” highlighting their professional struggles and lack of protection [5]. Similarly, a content analysis of physician-related news on Sina, one of China’s largest online news portals, revealed that, in 2016, portrayals of physicians as victims accounted for 27.23% of all medical news coverage, second only to reports portraying physicians in a positive light [6]. Representative articles include commentaries such as “We Should Not Learn from Doctors Who Die of Overwork” [7], which criticizes unrealistic expectations and highlights that >90% of physicians regularly lose sleep, or investigative reports such as “Doctor in Wenzhou Dies After Knife Attack; Hospital Releases Official Statement at Dawn” [8], which documents incidents of violence against medical staff. These examples further illustrate the growing visibility of vulnerability-themed portrayals in Chinese media narratives surrounding the medical profession.

The portrayal of physicians as vulnerable professionals is not confined to China; similar narratives are prevalent worldwide, underscoring systemic challenges in health care systems. In India, nearly 75% of physicians report experiencing workplace violence, and high-profile cases include the deaths of Dr Deben Dutta and Dr Vandana Das [9]. In the United States, physician burnout has increased, with a 2022 study revealing that 62.8% of physicians reported symptoms of burnout, a substantial increase from previous years. This surge is attributed to factors such as excessive workloads and emotional exhaustion. Media outlets have highlighted these concerns, noting that 86% of physicians fear for the future of their profession because of overwork and inadequate compensation [10]. In Australia, workplace violence against health care workers has also increased sharply; reports indicate a 48% increase in nurse assault cases in Queensland over a 3-year period, reflecting the broader risks faced by frontline medical professionals [11]. Despite the increasing visibility of these portrayals, their impact on public trust and perceptions is poorly understood.

The concept of vulnerability in news portrayals often lacks clear positive or negative attributes; instead, it is ambivalent and open to interpretation depending on viewers’ experiences and perceptions of the medical profession. Unlike portrayals that clearly present physicians as heroic (eg, saving lives during a crisis) or problematic (eg, involved in malpractice), vulnerable portrayals typically depict physicians as overworked, having inadequate compensation, or being victims of violence but do not directly state whether the audience should feel admiration, pity, or concern. As a result, these portrayals can send mixed messages. Some viewers may feel empathy and see physicians as self-sacrificing and caring [6,12], whereas others may question physicians’ ability to remain competent and authoritative under pressure, especially when such portrayals conflict with the traditional portrayal of the physician as a calm and authoritative figure [13]. For example, a fatigued physician shown resting between surgeries may be seen by some as dedicated and selfless, whereas others may interpret the same portrayal as unprofessional or concerning.

This ambiguity in how vulnerability is perceived highlights the need to examine such portrayals more closely in health care media research, particularly in light of their potential to shape public evaluations and trust. According to the stereotype content model, individuals evaluate others based on competence and warmth, which are crucial in shaping societal stereotypes and trust [14]. Warmth reflects perceived intentions, whereas competence relates to the ability to fulfill those intentions. Recent studies suggest separating morality from warmth, particularly in ethically charged professions such as medicine [15,16]. Therefore, this study focused on how vulnerable portrayals of physicians influence public evaluations across warmth, morality, and competence.

The explanatory framework adopted in this study to understand how media portrayals influence attitude change is based on the elaboration likelihood model (ELM), a widely used theory in social and media psychology proposed by Petty et al [17]. The ELM posits that persuasive messages are processed through either a central route, which involves careful evaluation of arguments, or a peripheral route, which relies on simple cues such as tone, visuals, or the messenger’s appeal. Involvement—how personally relevant the issue is to the audience, which, in this study, specifically refers to the audience’s personal relevance and connection to the medical issue being depicted—is a key factor influencing one’s motivation to process information. The ELM suggests that involvement shapes how much cognitive effort people are willing to devote and, thus, plays a critical role in attitude change [17].

According to the ELM, people with low involvement levels—those who do not care much about the issue or do not have personal experience with it—tend to use peripheral processing. They do not think too hard about the message but rely on surface-level cues such as how the message looks or who delivers it [17]. While some research suggests that source cues can still have a persuasive impact under low involvement, in the case of ambiguous media portrayals such as those of vulnerable physicians, the lack of clear evaluative cues may limit the formation of strong attitudes; the message may not offer clear signs about whether the audience should feel admiration or concern [18]. For people who are not involved in medical topics, this lack of clarity can make it difficult for them to form strong opinions because they do not care. Therefore, these portrayals might not significantly alter the evaluations of individuals with low involvement levels. Hence, hypothesis 1 is that, for individuals with low involvement levels, vulnerable physician media portrayals do not significantly impact warmth evaluations (hypothesis 1a), competence evaluations (hypothesis 1b), morality evaluations (hypothesis 1c), and overall evaluations (hypothesis 1d) of physicians.

For individuals with moderate involvement levels, the content of vulnerable physician portrayals is more likely to be processed elaborately; that is, such individuals pay closer attention to the message, reflect on its meaning, and connect it to previous experiences. These individuals might have some medical experiences, for example, visiting the hospital occasionally for themselves or their families, and, thus, feel more connected to the topic. They are more likely to think carefully about the content. Thus, they often feel in a subtly positive way and empathy is evoked in them when such portrayals highlight the challenges faced by physicians [5,19]. When viewing portrayals of physicians facing hardships, these individuals might notice the underlying positive tone, such as the physicians’ dedication, perseverance, or emotional strength. For example, a report describing a physician staying overnight to care for patients despite having a low income may prompt these viewers to perceive the physician as compassionate and committed to their duty. In contrast, viewers with low involvement levels—who lack interest in or a perceived relevance of medical topics—may process such information more superficially (eg, “This is just a story about an overworked and underpaid doctor, but it has nothing to do with me”). As a result, the impact on their evaluations tends to be weaker or even neutral.

Greater involvement increases favorable attitudes toward nuanced messages [18,20]. Thus, moderate involvement may enhance positive evaluations of physicians across dimensions such as competence, warmth, and morality, leading to improved trust in the medical profession. Hence, hypothesis 2 is that, for individuals with moderate involvement levels, vulnerable physician media portrayals positively impact their warmth evaluations (hypothesis 2a), competence evaluations (hypothesis 2b), morality evaluations (hypothesis 2c), and overall evaluations (hypothesis 2d) of physicians.

However, when individuals have high involvement levels, such as patients with chronic illnesses who frequently see physicians, they may already hold strong beliefs about what physicians should be like—confident, decisive, and in control. News portrayals showing physicians as vulnerable because they are overworked, anxious, or powerless may clash with these beliefs, causing discomfort or even rejection of the message. This phenomenon is known as cognitive dissonance, where conflicting thoughts create tension [21]. For example, a long-term patient who deeply respects their physician might feel uneasy seeing a media story showing that the physician is crying or being unsure. Instead of increasing empathy, this story could lead the viewer to think that the physician is weak or less competent.

In addition, portrayals that emphasize low power and status could further reinforce negative stereotypes about physicians’ competence and warmth [22]. For example, news stories that show physicians being scolded by patients’ families, being forced to work long hours in overcrowded conditions, or being the focus of unfair media accusations without defense can signal diminished social standing and professional power. Such portrayals may lead viewers to question physicians’ competence; diminish perceptions of their warmth; and, ultimately, erode trust in the medical profession. Therefore, ironically, for individuals with high involvement levels, such portrayals may provoke negative reactions as the portrayals clash with the individuals’ idealized expectations of physicians and create psychological discomfort rather than fostering empathy. Hence, hypothesis 3 is that, for individuals with high involvement levels, vulnerable physician media portrayals negatively impact their warmth evaluations (hypothesis 3a), competence evaluations (hypothesis 3b), morality evaluations (hypothesis 3c), and overall evaluations (hypothesis 3d) of physicians.

In summary, we hypothesize that vulnerable physician portrayals impact evaluations differently based on audience involvement. For those with low involvement levels, the portrayals may have little effect. For those with moderate involvement levels, they may strengthen positive evaluations. For highly involved individuals, the portrayals may contribute to negative evaluations. These hypotheses suggest an inverted U–shaped relationship in which the influence of vulnerable portrayals on evaluations is positive as involvement levels shift from low to moderate but becomes negative as involvement levels move from moderate to high.

Importantly, this study does not examine direct physician-patient interactions. Rather, it investigates how news portrayals of physicians shape public perceptions, which are socially constructed through media exposure rather than personal clinical experience, and how these perceptions influence trust in the medical profession. Trust is a foundational concept in social and organizational psychology and is generally defined as a psychological state involving the willingness to accept vulnerability based on positive expectations of another’s intentions or behavior [23]. Across disciplines, trust is understood as a multidimensional construct that includes competence (the ability to perform tasks effectively), benevolence (the belief that the trustee has good intentions), and integrity (adherence to a set of principles considered acceptable by the truster) [24,25]. Trust is greater when the target of trust (ie, the trustee)—in this context, the physician—is perceived as highly competent, benevolent, and principled [26]. For instance, in marketing, the combination of high warmth and competence enhances consumer trust and loyalty [27,28]. Given the unique nature of the medical profession, public trust in physicians depends not only on technical skill but also on perceptions of moral character and relational intent [25]. Patients are more likely to trust physicians who are perceived as highly competent, morally upright, and warm [29], making overall evaluation—a composite perception of competence, warmth, and morality—a critical precursor to trust. Accordingly, we hypothesize the following: the overall evaluation of physicians positively predicts their trust (hypothesis 4), and the overall evaluation of physicians mediates the relationship between vulnerable portrayal and trust in physicians (hypothesis 5).

**Figure 1:**
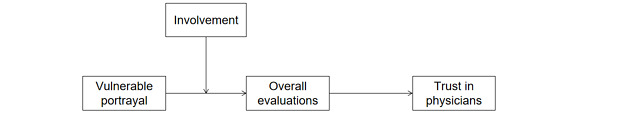
Hypothesis model.

The hypothesized model of this study is shown in Figure 1.

### Objectives

We conducted 4 studies to investigate how vulnerable news portrayals of physicians influence individuals’ evaluations and how this effect is moderated by involvement levels.

Studies 1 and 2 measured involvement using self-reported frequencies of hospital visits, which reflect a relatively stable and enduring level of engagement with the medical domain. Study 1 examined the relationship between exposure to vulnerable physician portrayals and evaluations of physicians among individuals with varying hospital visit frequencies. Study 2 further explored this issue by asking participants with different involvement levels to read a news article portraying physicians as vulnerable and then assessing its impact on their evaluations and trust in physicians.

Studies 3a, 3b, and 4 targeted individuals who reported hospital visits as *almost never*, *seldom*, or *moderately*, who constitute most of the general population. For these individuals, we hypothesized and found that involvement positively moderated the effect of vulnerable physician portrayals on evaluative outcomes. Accordingly, studies 3a and 3b tested whether situational involvement, when experimentally manipulated, could enhance the impact of vulnerable portrayals on evaluations of and trust in physicians. Study 4 embedded involvement-enhancing content within the vulnerable portrayal to test intervention strategies with greater ecological validity.

## Study 1

Involvement is rooted in personal interaction and engagement over time, reflecting a deep-seated interest in and concern about health-related interactions and decisions. Study 1 used a web-based questionnaire among individuals with varying involvement levels (ie, individuals with different frequencies of hospital visits) to examine the relationships among the frequency of exposure to news portraying vulnerable physicians, evaluations of physicians, and trust in physicians.

### Methods

#### Participants and Procedure

Participants were recruited through Credamo (an online research platform widely used in China) and were presented with specific attention-check questions at different stages (Multimedia Appendix 1). Responses from the participants who failed the attention checks were automatically marked as invalid. A total of 497 valid questionnaires were collected for the study, consisting of 161 (32.4%) male and 336 (67.6%) female participants with an average age of 31.70 (SD 8.63) years.

#### Ethical Considerations

Ethics approval was obtained from the Ethics Subcommittee of the Institute of Psychology, Chinese Academy of Sciences (H19050). Informed consent was obtained from all participants before their participation in the survey. Participants were provided with a detailed informed consent form outlining the purpose of the study, the duration of the investigation, the procedures, and the potential risks and benefits. They were informed that their participation was voluntary and that they had the right to withdraw at any time without facing any negative consequences. Participants were assured of the confidentiality and anonymity of their responses. Participants who effectively completed the survey in study 1 were given 7 RMB (US $0.98), participants in study 2 and 3 were compensated with 15 RMB (US $2.09), and participants in study 4 received 10 RMB (US $1.39). 

#### Measures

All the items were rated on 5-point Likert-type scales (1=*strongly disagree*; 5=*strongly agree*).

##### Regular Exposure to News Portraying Vulnerable Physicians

On the basis of the definition of vulnerable physician portrayals [5], the participants were asked about their exposure frequency to three news types in the previous 2 years: (1) reports about malicious violence against physicians or medical disputes, (2) reports highlighting the hardships and pressures faced by physicians in their work, and (3) reports highlighting poor compensation and limited career prospects for physicians. The Cronbach α coefficient was 0.63.

##### Involvement

Hospital visit frequency over the previous 2 years was used to measure enduring involvement and was scored from 1 (*almost never*) to 5 (*very frequently*). For the analysis, the participants were categorized into 3 groups: those who selected *almost never* (26/497, 5.2%) or *seldom* (181/497, 36.4%) were placed in the low-involvement group; those who selected the midpoint, *moderately* (171/497, 34.4%), were placed in the moderate-involvement group; and those who selected *more frequently* (97/497, 19.5%) or *very frequently* (17/497, 3.4%) were placed in the high-involvement group.

##### Evaluation

The stereotype content model questionnaire by Cheng and Guan [30] was used. For the warmth dimension, the Cronbach α coefficient was 0.80; for the competence dimension, it was 0.67; and, for the morality dimension, it was 0.69. The overall Cronbach α coefficient for the scale was 0.84.

##### Trust in Physicians

We adopted the patient trust scale [31], which consists of 4 items measuring how much a patient trusts a physician. We also administered 2 self-authored items: “Overall, I trust doctors and Doctors’ medical skills are not as good as they should be.” Higher total scores indicated greater trust in physicians. The Cronbach α coefficient for all the items was 0.83.

##### Control Variables

Demographic variables such as gender, age, educational level [32], average household monthly income [5], and personal health status can impact trust [33,34]; thus, these variables were included as covariables.

In this research, 2-tailed *t* tests and ANOVAs for all studies were conducted using the *BruceR* package (version 0.8.9) in R (version 3.3.0; R Foundation for Statistical Computing). In addition, other basic analyses were conducted using SPSS (version 26.0; IBM Corp). The bootstrap method was used for all studies using the Process plug-in in SPSS (version 26.0).

### Results

#### Correlations

In addition to being unrelated to evaluations of medical competence, regular exposure to vulnerable physician portrayals was significantly positively correlated with warmth toward physicians (*r*=0.15; *P*<.001), morality toward physicians (*r*=0.13; *P*=.005), overall evaluations (*r*=0.14; *P*=.001), and trust in physicians (*r*=0.15; *P*<.001). This result suggests that frequent exposure to vulnerable physician portrayals in everyday life might have a positive impact overall. In addition, evaluations of physicians and trust in physicians were significantly positively correlated (*r*=0.82; *P*<.001), providing preliminary support for hypothesis 4. Multimedia Appendix 1 provides more details.

#### Regression Models

We used the participants’ regular exposure to vulnerable physician portrayals as the independent variable and evaluations, including their subdimensions, and participants’ trust in physicians as the dependent variables. After controlling for other variables, we conducted group-based regressions. Using hierarchical regression and considering the moderate-involvement group as the reference group, we subsequently calculated the significant differences in coefficients between the low- and moderate-involvement groups and between the moderate- and high-involvement groups.

The results, as presented in Table 1, reveal that regular exposure to vulnerable physician portrayals positively predicted warmth toward physicians and overall evaluations in the low-involvement group; thus, hypotheses 1a and 1d were not supported. Within the moderate-involvement group, regular exposure to vulnerable physician portrayals significantly marginally predicted evaluations of medical competence and significantly predicted participants’ warmth, moral evaluations, overall evaluations, and trust in physicians, thus supporting hypotheses 2a to 2d. Unlike hypotheses 3a to 3d, regular exposure to vulnerable physician portrayals did not significantly predict any dependent variables in the high-involvement group. Nonetheless, the predictive effect of exposure to vulnerable physician portrayals on moral evaluation was significantly stronger in the moderate-involvement group than in the low-involvement group, whereas the coefficients for other indicators, although not significantly different, were also greater than those in the low-involvement group. As the enduring involvement level subsequently increased, the predictive coefficients of frequent exposure to vulnerable physician portrayals became significantly lower for all the dependent variables in the high-involvement group than for those in the moderate-involvement group except for competence evaluations. These trends support our hypothesis of an inverted U–shaped moderation effect of involvement level on the impact of vulnerable physician portrayals.

**Table 1:** The coefficients of the grouped regression models of study 1^a^.

Dependent variable	Low involvement (n=207)			Moderate involvement (n=171)			High involvement (n=114)			Moderate-low			Moderate-high	
	*B* (SE)	*P* value	*B* (SE)		*P* value	*B* (SE)		*P* value	*t* test (*df*)		*P* value	*t* test (*df*)		*P* value
Warmth	0.18 (0.07)	.01	0.24 (0.07)		.001	0.03 (0.07)		.68	0.79		.43	1.97		.05
Competence	0.08 (0.06)	.15	0.11 (0.06)		.07	−0.01 (0.06)		.81	0.67		.50	1.49		.14
Morality	0.07 (0.06)	.25	0.25 (0.06)		<.001	−0.02 (0.06)		.76	2.05		.04	2.98		.003
Evaluations	0.11 (0.05)	.04	0.20 (0.05)		<.001	0.00 (0.06)		.97	1.40		.16	2.59		.01
Trust	0.15 (0.06)	.006	0.18 (0.06)		.003	−0.00 (0.05)		.97	0.50		.62	2.29		.02

^a^The independent variable is the frequency of exposure to vulnerable physician portrayals in everyday life. *Moderate-low* refers to the comparison of regression coefficients between the moderate- and low-involvement groups, assessing the differences in the predictive effect of exposure to vulnerable physician portrayals on each dependent variable. Similarly, *moderate-high* refers to the comparison of regression coefficients between the moderate- and high-involvement groups.

After the demographic variables were controlled for, model 7 was chosen for the mediation analysis, with involvement level as the moderating variable. The moderate-involvement group was set as the reference group (coded as 000), and 5000 samples were selected within a 95% CI. The results are presented in Multimedia Appendix 1. For the moderate-involvement group, regular exposure to portrayals of vulnerable physicians in everyday life was found to positively predict evaluations. In addition, evaluations were found to further positively predict trust in physicians (*b*=0.86; *P*<.001), thus supporting hypothesis 4. When the moderate-involvement group was used as the reference group, only the moderating effect of high involvement was significant (*b*=−0.19; *P=*.01). A further simple-slope analysis revealed that, for the low-involvement group, the positive predictive effect of exposure to vulnerable physician portrayals on overall evaluations was marginally significant (simple slope=0.10; SE 0.05; *P*=.05) and the evaluations mediated the relationship between exposure to vulnerable physician portrayals and trust in physicians; the indirect effect was 0.08 (95% CI 0.002-0.17). For the moderate-involvement group, this positive predictive effect was strengthened (simple slope=0.20; SE 0.05; *P*<.001), and the indirect effect was 0.17 (95% CI 0.05-0.29; Multimedia Appendix 1), thereby supporting hypothesis 5. However, for the participants in the high-involvement group, the predictive effect of exposure to vulnerable physician portrayals on the evaluations was no longer significant (simple slope=0.003; SE 0.05; *P*=.95).

### Discussion

Overall, this study revealed that regular exposure to vulnerable physician portrayals was positively correlated with individuals’ warmth evaluations, moral evaluations, overall evaluations, and trust in physicians. This result implies a positive impact stemming from prolonged exposure to such neutral, vulnerable physician media portrayals. However, this positive correlation varied across individuals with different involvement levels. Specifically, as involvement increased from low to moderate, the positive correlation strengthened between exposure to vulnerable portrayals and evaluations. Conversely, as involvement increased from moderate to high, the positive correlation between exposure and evaluations weakened. The correlations between vulnerable portrayals and evaluations, as well as trust in physicians, exhibited similar inverted U–shaped changes with increasing involvement. Study 2 further investigated the causal effects of viewing vulnerable physician portrayals on evaluations and trust in physicians among individuals with different involvement levels.

## Study 2

### Methods

#### Overview

Study 2 manipulated the participants by having them directly read news articles portraying physicians as vulnerable. This study aimed to causally explore the impact of reading vulnerable physician portrayals on participants’ evaluations of and trust in physicians based on the individuals’ varying involvement levels. In addition, study 1 did not confirm the negative effects of exposure to vulnerable physician portrayals under high involvement levels. We speculated that this might be due to the limited highest level of involvement represented in the online sample recruited from everyday contexts. To address this issue, we collected data from participants in an environment with superhigh involvement levels, that is, a hospital setting.

While we acknowledge that the combination of recruitment methods—participants from specific hospital departments versus the general Credamo sample—may introduce confounding factors, we believe that it is crucial to collect data from patients visiting hospitals when studying the impact of vulnerable physician portrayals on public evaluations of and trust in physicians. By intentionally selecting 2 departments in which patients are likely to require multiple visits, we ensured that our sample represented individuals with superhigh involvement levels in their medical care.

#### Participants

A total of 129 participants (n=49, 38% male and n=80, 62% female; mean age 46.67, SD 17.38 years) were recruited from the ultrasound department and the acupuncture department of 2 tertiary hospitals in Beijing. These individuals, either patients or their accompanying family members, were classified into the *superhigh-involvement group* because of their frequent and ongoing interactions with the health care system, which indicated a superhigh level of involvement and engagement with medical care.

The remaining participants were recruited from the general population through Credamo. Initially, 366 valid responses were collected, but moderate- (n=76, 20.8%) and high-involvement (n=27, 7.4%) groups were underrepresented. To address this problem, we distributed 550 screening questionnaires and specifically targeted those with moderate or high hospital visit frequencies, ultimately obtaining the additional responses needed.

All the participants (270/710, 38% male and 440/710, 62% female; mean age 34.47, SD 11.57 years) were categorized into 4 involvement groups (low: 307/710, 43.2%; moderate: 161/710, 22.7%; high: 113/710, 15.9%; superhigh: 129/710, 18.2%) and were randomly assigned to read 1 of 2 news articles (vulnerable portrayal vs control). Cell sample sizes ranged from 55 to 160 (“cells” refers to the 8 groups in the 2 × 4 between-subjects design of the experiment; the sample sizes for these groups ranged from 55 to 160 participants).

#### Procedure

The participants were randomly assigned to read either an article depicting physicians as vulnerable (which reflected the 3 most commonly referenced aspects of vulnerable physician portrayals in Chinese media: long working hours, low income, and strained relationships with patients; Multimedia Appendix 1) or a control article about a person looking for a lost backpack. To enhance ecological validity, the vulnerable physician article was adapted from real-world news coverage [35]. Following the reading, only those in the vulnerable physician article group answered a question to confirm the article’s impact. The control content, which was similar in format and length but lacked emotional depth, required no manipulation check. After reading the news, the participants were asked to complete the questionnaire.

#### Measures

##### Manipulation Check for Vulnerable Portrayals

The participants in the experimental group were asked to rate the portrayed physician on a scale from 1 (*very vulnerable*) to 5 (*very powerful*). A 1-sample *t* test was conducted to compare the participants’ perceptions of the news portrayals, with a median score of 3 (IQR 2-4). The results revealed that the participants perceived the physician portrayal in the news as significantly leaning toward vulnerability (mean 1.56, SD 0.61; *t*_139_=−27.77; *P*<.001), confirming the successful manipulation of a vulnerable physician news portrayal.

##### Involvement

Involvement measures were the same as those for study 1.

##### Evaluation Scale

As in study 1, the Cronbach α was 0.83 for warmth, 0.70 for competence, 0.78 for morality, and 0.87 for the overall scale.

##### Patient Trust Scale

As in study 1, the Cronbach α was 0.74.

##### Control Variables

The control variables included gender, age, educational level, monthly income, and physical health, as in study 1. In addition, based on the results of study 1, the participants’ regular exposure to vulnerable physician portrayals needed to be controlled. To minimize the reading burden on the participants, we measured only the participants’ frequency of reading malicious physician-related news (“Please indicate how often you have paid attention to and read news about malicious incidents involving harm to doctors in the past two years”; 1=*never paid* attention; 5=*always paid attention*).

### Results

#### 2-Way ANOVA

We first analyzed all the evaluations via a 2 (portrayal—vulnerable vs control) × 4 (involvement—low vs moderate vs high vs superhigh) 2-way ANOVA. The results are presented in Table 2.

**Table 2:** Results of the 2-way ANOVA for study 2.

Dependent variable	Main effect of portrayal				Main effect of involvement				Interaction effect		
	*F* test (*df*)	*P* value	η_p_^2^	*F* test *(df)*		*P* value	η_p_^2^	*F* test *(df*)		*P* value	η_p_^2^
Warmth	0.333 (1, 702)	.56	0.000	11.584 (3, 702)		<.001	0.047	4.261 (3, 702)		.005	0.018
Competence	0.317 (1, 702)	.57	0.000	14.933 (3, 702)		<.001	0.060	1.778 (3, 702)		.15	0.008
Morality	0.293 (1, 702)	.59	0.000	4.388 (3, 702)		.005	0.018	1.060 (3, 702)		.37	0.005
Evaluations	0.447 (1, 702)	.50	0.001	13.804 (3, 702)		<.001	0.056	3.016 (3, 702)		.03	0.013
Trust	2.008 (1, 702)	.16	0.003	6.193 (3, 702)		<.001	0.026	0.229 (3, 702)		.88	0.001

For the dependent variables of perceived physician evaluations and their dimensions as well as physician trust, the main effect of the weak portrayal manipulation was not significant for total physician evaluations or their 3 dimensions. However, the main effect of involvement level was significant for overall evaluations and their 3 dimensions. Post hoc test results are shown in Multimedia Appendix 1, revealing that the superhigh-involvement group rated overall evaluations and their dimensions significantly more positively than the high-, moderate-, and low-involvement groups. Except for the dimension of morality, the high- and moderate-involvement groups rated overall evaluations, as well as the dimensions of warmth and competence, significantly more positively than the low-involvement group.

The interaction effect between vulnerable portrayal and involvement level was significant for overall evaluations and the warmth dimension. A further simple-effects analysis was conducted with total evaluations and each dimension as dependent variables (Table 3). For individuals with low involvement levels, there was no significant difference in evaluations between the vulnerable portrayal group and the control group (*t*<0.16 in all cases; *P*>.15 in all cases), supporting hypotheses 1a~1d. For individuals with moderate involvement levels, the vulnerable portrayal group yielded marginally better evaluations than the control group (*t*_702_=1.67; *P*=.10; *d*=0.26), supporting hypothesis 2d. For individuals with high involvement levels, there was no significant difference in evaluations between the 2 groups (*t*<0.49 in all cases; *P*>.62 in all cases). However, for the superhigh-involvement group within the hospital setting, the vulnerable portrayal group evaluated physicians significantly worse in terms of warmth, competence, and overall evaluations than the control group (overall evaluations: *t*_702_=−2.49, *P*=.01, and *d*=−0.44; warmth: *t*_702_=−3.15, *P*=.002, and *d*=−0.56; competence: *t*_702_=−1.70, *P*=.09, and *d*=−0.30), supporting hypotheses 3a, 3b, and 3d.

**Table 3:** Evaluation scores of physicians for each group in study 2.

		Warmth (score of 1-5), mean (SD)	Competence (score of 1-5), mean (SD)	Morality (score of 1-5), mean (SD)	Overall evaluations (score of 1-5), mean (SD)	Trust (score of 1-5), mean (SD)
**Low involvement**						
	Control	3.63 (0.80)	4.19 (0.57)	4.05 (0.66)	3.96 (0.56)	3.70 (0.59)
	Vulnerable portrayal	3.65 (0.80)	4.13 (0.68)	3.97 (0.73)	3.91 (0.59)	3.73 (0.65)
**Moderate involvement**						
	Control	3.68 (0.69)	4.16 (0.54)	4.11 (0.57)	3.98 (0.49)	3.81 (0.59)
	Vulnerable portrayal	3.89 (0.62)	4.27 (0.43)	4.26 (0.54)	4.14 (0.41)	3.94 (0.56)
**High involvement**						
	Control	3.75 (0.78)	4.27 (0.62)	4.28 (0.57)	4.10 (0.50)	3.96 (0.54)
	Vulnerable portrayal	3.83 (0.78)	4.25 (0.42)	4.29 (0.54)	4.12 (0.44)	3.99 (0.55)
**Superhigh involvement**						
	Control	4.36 (0.99)	4.47 (0.92)	4.57 (0.82)	4.46 (0.85)	3.81 (0.60)
	Vulnerable portrayal	3.91 (0.94)	4.33 (0.87)	4.37 (0.89)	4.20 (0.83)	3.88 (0.63)

When trust was taken as the dependent variable, the results indicated that only the main effect of involvement level was significant. Post hoc tests revealed that trust in physicians was marginally greater in the high-involvement group than in the superhigh-involvement group (*t*_702_=1.68; *P*=.09; *d*=0.22). In addition, trust was significantly greater in the superhigh- (*t*_702_=2.09; *P*=.04; *d*=0.22), high- (*t*_702_=3.96; *P*<.001; *d*=0.44), and moderate-involvement (*t*_702_=2.72; *P*=.004; *d*=0.27) groups than in the low-involvement group.

#### Regression Analysis

Similarly, the Process plug-in in SPSS was used, and model 7 was selected. A regression analysis was conducted with vulnerable portrayals as the independent variable and involvement level grouping as the moderator (Multimedia Appendix 1). When the moderate-involvement group was used as the reference, the interaction effects of low and superhigh involvement were both significant. A further simple-slope analysis indicated that vulnerable portrayals had a marginally positive effect on the evaluations of medical professionals only for individuals with moderate involvement levels (simple slope=0.16; SE 0.09; *P*=.10), which further positively influenced their trust in physicians (*b*=0.54; *P*<.001); the indirect effect was 0.08 (95% CI 0.01-16), once again supporting hypotheses 4 and 5. However, for individuals with low (simple slope=−0.05; SE 0.07; *P*=.47) and high (simple slope=0.03; SE 0.11; *P*=.82) involvement levels, vulnerable portrayals did not have a significant effect on evaluations. For the superhigh-involvement group in the hospital, vulnerable portrayals had a significant negative predictive effect on overall evaluations (simple slope=−0.23; SE 0.11; *P*=.03). However, overall evaluations did not have a significant negative indirect effect on the relationship between vulnerable portrayals and trust (Multimedia Appendix 1).

### Discussion

Study 2 used an experimental design to explore how news portrayals of vulnerable physicians affect individuals with differing involvement levels. The results indicated that exposure to such portrayals did not significantly alter the evaluations or trust of the general population except in individuals with moderate involvement levels, who experienced positive shifts in evaluations, indirectly boosting their trust. These outcomes reinforce the notion that the impact of news portrayals of vulnerable physicians on public perceptions is influenced by the degree of individual involvement.

In addition, the study revealed that vulnerable physician portrayals negatively impacted the evaluations of physicians among patients or their relatives, especially those in departments dealing with chronic conditions, signifying individuals’ very high involvement levels. This result suggests the detrimental effect of such portrayals on patients with a deep, ongoing focus on health issues, highlighting the need to avoid pushing vulnerable physician portrayals in medical settings. Interestingly, those with the highest involvement levels yielded more positive evaluations than the public, possibly due to the former’s extensive medical experiences and the influence of self-perception theory [21], suggesting that their proactive health care behavior leads to a favorable view of physicians.

During data collection, only 23.2% (114/492) of study 1’s participants had high involvement levels, decreasing to 7.4% (27/366) in study 2’s initial data collection phase among those not seeking medical care. For these individuals, the impact of vulnerable physician portrayals on evaluations appeared to improve with increased involvement. Consequently, this study focused on the majority with low to moderate involvement levels, investigating whether increasing their involvement could increase the positive effects of media portrayals of vulnerable physicians.

## Study 3

### Overview

In the first 2 studies, involvement was operationalized as hospital visit frequency, reflecting a relatively stable and enduring level of engagement with the medical domain. This form of involvement, referred to as enduring involvement, is often shaped by personal health status or long-term life circumstances and, therefore, is difficult to modify through short-term interventions [36].

To examine causal mechanisms and inform scalable interventions, it is important to consider situational involvement, which is a more temporary, context-dependent form of engagement that can be influenced by task relevance or external stimuli [36]. Importantly, situational involvement may function independently of enduring involvement and offers greater flexibility in experimental settings [37].

Given that studies 1 and 2 showed positive moderating effects of involvement among individuals who reported *almost never* to *moderate* hospital visits, a group that constituted >70% of our sample (study 1: 378/492, 76.8%; study 2: 339/366, 92.6%), studies 3 and 4 specifically targeted this population. In this study, we sought to manipulate situational involvement by enhancing the participants’ perceived relevance of a health care scenario in the immediate context. To achieve this goal, we used an imagination-based paradigm asking the participants to vividly imagine themselves in a medical consultation scenario. This approach induces mental simulation, allowing individuals to experience a context cognitively without requiring real-life exposure. Previous research has shown that imagination exercises can effectively enhance perceived personal relevance and engagement [38,39], particularly in health communication contexts, without causing excessive reactance or overinvolvement, which may occur in high-stakes real-world settings. Compared with direct exposure in clinical environments, imagined scenarios offer a moderate and controlled increase in involvement, making them well suited for the experimental testing of psychological mechanisms.

We propose that, for most individuals—those with low to moderate enduring involvement levels (ie, those who almost never, seldom, or moderately visit hospitals)—a moderate increase in situational involvement through imagination may improve their responsiveness to news portrayals of vulnerable physicians. On the basis of this rationale, hypothesis 6 is that, under low situational involvement, reading vulnerable physician portrayals does not significantly influence the warmth evaluations (hypothesis 6a), competence evaluations (hypothesis 6b), morality evaluations (hypothesis 6c), or overall evaluations (hypothesis 6d) of individuals with low to moderate enduring involvement levels (ie, those who almost never, seldom, or moderately visit hospitals), and hypothesis 7 is that, under high situational involvement, reading vulnerable physician portrayals positively influences the warmth evaluations (hypothesis 7a), competence evaluations (hypothesis 7b), morality evaluations (hypothesis 7c), and overall evaluations (hypothesis 7d) of individuals with low to moderate enduring involvement levels (ie, those who almost never, seldom, or moderately visit hospitals).

In total, 2 substudies were conducted: study 3a used a questionnaire to manipulate situational involvement, whereas study 3b used a more detailed laboratory experiment with an added no-involvement group to examine the effects on perceptions of vulnerable physician portrayals.

### Study 3a

#### Methods

Study 3a used a 2 (portrayals—vulnerable vs control) × 2 (situational involvement—high vs low) between-subject design and was conducted through an online questionnaire. The dependent variables included the participants’ evaluations of and trust in the physician depicted in the materials.

##### Participants

The required sample size was estimated using G*Power (version 3.1) [40] to achieve 95% power for the main effect test with an effect size of 0.25. The analysis indicated that the total required sample size was 279. The participants were recruited through both the Questionnaire Star and Credamo platforms. Ultimately, 497 valid questionnaires were collected. Among them, only 81.5% (405/497) of the participants, whose hospital visit frequency ranged from *almost never* to *moderate*, were retained as they represented the target population of this study (151/405, 37.3% male and 254/405, 62.7% female; mean age 31.03, SD 6.33 years). The participants were randomly assigned to 1 of 4 cells; cell sample sizes ranged from 95 to 111.

##### Procedure

The experimental procedure consisted of 3 main parts, which are outlined in the following sections.

#### Step 1

Drawing from Berger [41], this step manipulated situational involvement using second- and third-person narratives. The participants were randomly assigned to either the high- or low-involvement group and were presented with 1 of 2 scenario descriptions. First, high situational involvement; you have had a recurring fever for the last two months. You took antibiotics to eliminate the infection, but the fever returned today. Therefore, you go to a general clinic. The doctor asks you about your basic symptoms and then asks you to do a blood test before coming back, so you start lining up for the blood test. Second, low situational involvement; Mr Wang has had a recurring fever for the last two months. He took antibiotics to eliminate the infection, but the fever returned today. Therefore, Mr Wang went to a general clinic. The doctor asks him about his basic symptoms and then asks him to perform a blood test before coming back. Therefore, Mr Wang began to line up for the blood test.

To increase the manipulation effect, the participants in the high–situational involvement group were instructed to immerse themselves in the situation and briefly describe their thoughts or emotions at that moment. In contrast, the participants in the low–situational involvement group were asked only to briefly describe the weather.

#### Step 2

The participants were then asked to imagine themselves or Mr Wang encountering a news article while waiting for the test results. They were randomly assigned to read either a news article of a vulnerable physician media portrayal or a control news article. After reading the news article, the participants were instructed to complete the evaluation scale.

#### Step 3

The participants were presented with a scenario based on the following theme: “You have/Mr. Wang has a fever, and the doctor suggests a bone marrow aspiration.” This scenario material, adapted from the work by Lyu et al [31], was presented in a simplified comic format to increase readability (Multimedia Appendix 1). The participants were subsequently asked to complete the trust scale and report their demographic characteristics.

##### Measures

#### Evaluation Scale

As in study 1, the Cronbach α was 0.74 for warmth, 0.56 for competence, 0.70 for morality, and 0.76 for the overall scale.

#### Patient Trust Scale

The comprehensive patient trust measurement scale used by Lyu et al [31], comprising 13 items, was used. Item 8 was reverse scored. Higher total scores indicated higher trust levels in physicians, with a Cronbach α of 0.92.

#### Control Variables

As in study 2, the control variables included gender, age, educational level, monthly income, physical health, and general attention paid to news related to malicious medical injury incidents.

### Results

A 2 (portrayal—vulnerable vs control) × 2 (situational involvement—low vs high) ANOVA was conducted. The results are presented in Multimedia Appendix 1. The main effects of vulnerable portrayal and situational involvement were not significant for overall evaluations or their subdimensions. The interaction effect was significant only for the warmth dimension (*F*_1, 401_=4.64; *P*=.02) and overall evaluations (*F*_1, 401_=4.88; *P*=.03). A further simple-effect analysis indicated that, compared with those in the control group, individuals in the low–situational involvement group reading a vulnerable portrayal showed no significant differences in overall evaluations or their 3 dimensions (*t*>−0.85 in all cases; *P*>.39 in all cases), providing support for hypotheses 6a~6d. In addition, based on the trend observed in the results (Table 4), in the low–situational involvement condition, the scores in all 3 dimensions were lower in the vulnerable portrayal group than in the control group.

**Table 4:** Evaluation scores of physicians for each group in study 3a.

		Warmth (score of 1-5), mean (SD)	Competence (score of 1-5), mean (SD)	Morality (score of 1-5), mean (SD)	Overall evaluations (score of 1-5), mean (SD)	Trust (score of 1-5), mean (SD)
**Low involvement**						
	Control	3.74 (0.73)	4.22 (0.62)	4.11 (0.63)	4.02 (0.48)	3.55 (0.77)
	Vulnerable portrayal	3.64 (0.76)	4.23 (0.60)	4.09 (0.72)	3.99 (0.55)	3.66 (0.70)
**High involvement**						
	Control	3.52 (0.81)	4.15 (0.66)	4.00 (0.75)	3.89 (0.59)	3.49 (0.74)
	Vulnerable portrayal	3.76 (0.79)	4.32 (0.55)	4.18 (0.65)	4.09 (0.51)	3.68 (0.74)

For the participants in the high–situational involvement group, the vulnerable portrayal group’s evaluations were significantly more positive than those of the control group (overall evaluations: *t*_401_=2.71, *P*=.007, and *d*=0.37; warmth: *t*_401_=2.23, *P*=.03, and *d*=0.31; competence: *t*_401_=2.06, *P*=.04, and *d*=0.28; morality: *t*_401_=1.96, *P*=.05, and *d*=0.27), thus supporting hypotheses 7a~7d.

Regarding patient trust, the main effect of vulnerable portrayal was significant (*F*_1,401_=4.26; *P*=.04). The participants who read vulnerable portrayals showed greater trust in physicians (mean 3.68, SD 0.052) than those in the control group (mean 3.52, SD 0.052). The main effects of situational involvement and their interaction were not significant. However, a further simple-effect analysis revealed that, in the low–situational involvement group, individuals reading vulnerable portrayals showed no significant difference in trust compared with the control group (*t*_401_=1.04; *P*=.30). In the high–situational involvement group, individuals in the vulnerable portrayal group exhibited a marginally significant increase in trust compared with those in the control group (*t*_401_=1.90; *P*=.06; *d*=0.26).

A regression analysis was conducted with situational involvement group as the moderating variable (low and high situational involvement were coded as 0 and 1, respectively). The results are presented in Multimedia Appendix 1. The moderating effect of situational involvement was significant (*b*=0.23; *P*=.03). A further simple-slope analysis was conducted. In the low–situational involvement condition, vulnerable portrayal exhibited a nonsignificant negative predictive trend for evaluations (simple slope=−0.04; SE 0.07; *P*=.57). In the high–situational involvement condition, vulnerable portrayal had a significant positive predictive effect on evaluations (simple slope=0.18; SE 0.07; *P*=.009), which further positively influenced trust in physicians (*b*=0.71; *P*<.001); the indirect effect was 0.13 (95% CI 0.03-24), supporting hypotheses 4 and 5.

### Discussion

Study 3a used a questionnaire-based approach to validate the impact of vulnerable physician portrayals on evaluations and patient trust among individuals with low to moderate frequencies of hospital visits (comprising 405/497, 81.5% of the general population in this experiment). By using an imaginative medical paradigm to increase situational involvement, this study demonstrated a positive moderating effect on the relationship between vulnerable physician portrayals and evaluations of physicians. This effect was further augmented by the indirect mediating effect of evaluations on patient trust. Moreover, study 3a revealed that, within the context of imagining an unrelated individual’s medical scenario (low–situational involvement group), viewing portrayals of vulnerable physicians had a consistent yet nonsignificant negative impact on evaluations, specifically in the warmth and morality dimensions. One possible explanation for this finding is that imagining a medical scenario unrelated to oneself may decrease personal situational involvement, thereby exacerbating the negative influence of vulnerable physician portrayals. To verify this hypothesis, we introduced a no-involvement group and conducted a more rigorous laboratory experiment.

### Study 3b

#### Methods

Study 3b used a 2 (portrayal—vulnerable vs control) × 3 (situational involvement—high vs low vs no involvement) between-subject design in a laboratory setting. Similarly to study 3a, study 3b used the same measurement tools and procedures but introduced a no-involvement group as a baseline control. An additional evaluation was conducted after manipulating situational involvement to isolate its effects. To explore the lasting impact of vulnerable physician portrayals, a 12-minute unrelated cognitive task followed the involvement activation and news reading.

We recruited 182 participants and retained 160 (87.9%) whose hospital visit frequency ranged from *almost never* to *moderate* (n=47, 29.4% male and n=113, 70.6% female; mean age 22.52, SD 2.45 years). They were randomly assigned to 1 of 6 conditions. All the measurement instruments used were consistent with those used in study 3a.

#### Results

Similarly to study 3a, study 3b revealed significant main effects of vulnerable portrayals on trust and overall evaluations and their 3 dimensions (*P*<.05 in all cases). Although the interaction effect between vulnerable portrayal and situational involvement was not significant for overall evaluations or their 3 dimensions (*F*_2,154_<2.10 in all cases; *P*>.12 in all cases), simple-effect tests revealed that, after high involvement, the vulnerable portrayal group scored significantly higher across all evaluations than the control group, supporting hypotheses 7a to 7d. In contrast, no significant differences were found between the low–situational involvement vulnerable portrayal group and the control group, supporting hypotheses 6a to 6d. Interestingly, without involvement manipulation, the vulnerable portrayal group yielded better moral and overall evaluations than the control group, but this positive impact was evident in the no-involvement group and not in the low–situational involvement group. This result suggests that associating a vulnerable physician portrayal with a medical situation involving unrelated others may have a negative effect. Detailed results are available in Multimedia Appendix 1.

Consistent with study 3a, a regression analysis further confirmed that high situational involvement positively moderated the relationship between vulnerable portrayals and evaluations, further enhancing trust and supporting hypotheses 4 and 5. No significant effects were found under the low–situational involvement or no-involvement conditions. Detailed results are available in Multimedia Appendix 1.

#### Discussion

In study 3b, a laboratory experiment involving participants with low to moderate levels of hospital visit frequency (160/182, 87.9% of the sample) used a medical scenario imagination paradigm to enhance situational involvement, which positively moderated the effects of vulnerable physician portrayals on evaluations and trust. Unlike study 3a, in which low situational involvement had a nonsignificant negative impact, study 3b revealed that positive effects on morality and overall evaluations were evident in the no-involvement group but not in the low–situational involvement group. This result suggests that associating vulnerable physician portrayals with medical scenarios involving unrelated others may have a negative effect. The results of both studies highlight the potential of situational involvement manipulation to enhance the positive perceptions of physicians, suggesting avenues for future research.

## Study 4

### Methods

#### Overview

Studies 3a and 3b showed that increasing situational involvement can positively moderate the impact of vulnerable portrayals among the majority with low to moderate enduring involvement (ie, hospital visit frequency). Study 4 tested a more ecologically valid intervention—embedding involvement-enhancing content directly into the news. Using a 1-factor between-subject design (control, vulnerable portrayal, and high-involvement vulnerable portrayal), the study tested whether prefacing a vulnerable portrayal with “medical matters concern everyone” can increase situational involvement and improve the evaluations of physicians.

Hypothesis 8 is that participants in the high-involvement vulnerable portrayal group will yield significantly higher physician warmth evaluations (hypothesis 8a), competence evaluations (hypothesis 8b), morality evaluations (hypothesis 8c), overall evaluations (hypothesis 8d), and trust ratings (hypothesis 8e) than those in the control group, and hypothesis 9 is that participants in the high-involvement vulnerable portrayal group will yield significantly higher physician warmth evaluations (hypothesis 9a), competence evaluations (hypothesis 9b), morality evaluations (hypothesis 9c), overall evaluations (hypothesis 9d), and trust ratings (hypothesis 9e) than those in the vulnerable portrayal group.

#### Participants

A total of 518 valid questionnaires were collected through Credamo. A total of 436 questionnaires from participants with low to moderate enduring involvement were subsequently retained (n=155, 35.6% control; n=140, 32.1% vulnerable portrayal; and n=141, 32.3% high-involvement vulnerable portrayal). The participants had an average age of 30.04 (SD 7.95) years, with 41.3% (180/436) being male and 58.7% (256/436) being female.

#### Procedure

The participants were randomly assigned to read a vulnerable physician portrayal news article with high situational involvement, a vulnerable physician portrayal news article, or a control group news article (Multimedia Appendix 1). After reading, the participants in both vulnerable news article groups were asked to answer manipulation check questions to assess whether the news successfully portrayed a vulnerable physician (similarly to study 2). In addition, all 3 groups were asked to respond to a manipulation check question about involvement (“How relevant do you think seeing a physician and seeking medical treatment is to you?”; 1=*not relevant at all*; 5=*very relevant*). After reading the news, the participants were asked to respond to the same measures as those used in study 2.

### Results

#### Manipulation of Test Results

A 1-sample *t* test was conducted for both of the experimental groups to compare the participants’ perceived portrayal, with a median score of 3. The results revealed that both groups perceived the portrayal to be significantly skewed toward vulnerability (vulnerable portrayal group: mean 1.56, SD 0.61, *t*_139_=−27.77, and *P*<.001; high-involvement vulnerable portrayal group: mean 1.57, SD 0.64, *t*_140_=−26.65, and *P*<.001). This result indicates the successful manipulation of the portrayal of vulnerable physicians.

A 1-way ANOVA was conducted to examine the perceived relevance of the medical scenario across the 3 groups. The results revealed a marginally significant difference among the groups (*F*_2,433_=2.83; *P*=.06). Post hoc comparisons indicated that the perceived relevance of seeking medical treatment in the high-involvement vulnerable portrayal group (mean 4.51) was significantly greater than that in the control group (mean 4.30; *t*_433_=2.37; *P*=.02; *d*=0.28), indicating a successful involvement manipulation in the high-involvement group. The vulnerable portrayal group (mean 4.41) demonstrated no significant differences from the other 2 groups (*P*>.22 in all cases).

#### ANOVA and Regression Analysis

A 1-way ANOVA was conducted with group as the independent variable and stereotypes as the dependent variables. The results (Multimedia Appendix 1) revealed significant main effects for the dimensions of warmth, morality, and overall evaluations, as shown in Table 5. Post hoc comparisons indicated that the participants in the high-involvement vulnerable portrayal group yielded significantly higher for warmth evaluations, morality evaluations, and overall evaluations than those in the control group (warmth: *t*_433_=2.99, *P*=.003, and *d*=0.35; morality: *t*_433_=2.78, *P*=.006, and *d*=0.32; overall evaluations: *t*_433_=3.14, *P*=.002, and *d*=0.37). In addition, although not statistically significant, evaluations of physicians’ competence were greater in the high-involvement vulnerable portrayal group than in the vulnerable portrayal group, partially supporting hypotheses 8a~8d.

**Table 5:** Evaluation scores of physicians for each group in study 4.

	Warmth (score of 1-5), mean (SD)	Competence (score of 1-5), mean (SD)	Morality (score of 1-5), mean (SD)	Overall evaluations (score of 1-5), mean (SD)	Trust (score of 1-5), mean (SD)
Control group	3.62 (0.74)	4.33 (0.63)	4.09 (0.65)	4.01 (0.55)	4.03 (0.64)
Vulnerable portrayal group	3.72 (0.65)	4.41 (0.46)	4.20 (0.51)	4.11 (0.41)	4.03 (0.60)
High-involvement vulnerable portrayal group	3.85 (0.63)	4.43 (0.53)	4.28 (0.55)	4.19 (0.45)	4.16 (0.53)

Furthermore, evaluations of physicians’ warmth in the high-involvement vulnerable portrayal group were marginally significantly higher than those in the vulnerable portrayal group (*t*_433_=1.70; *P*=.09; *d*=0.20), and competence evaluations, morality evaluations, and overall evaluations were numerically higher, providing partial support for hypotheses 9a~9d. In contrast, the vulnerable portrayal group scored only marginally higher than the control group (*t*_433_=1.73; *P*=.08; *d*=0.20). These findings indicate that increasing vulnerability through increased involvement can significantly improve the evaluations of participants with low to moderate enduring involvement compared with a simple portrayal of vulnerable medical professionals.

With trust in physicians as the dependent variable, the main effect was not significant. However, post hoc comparisons revealed a marginally significant increase in trust in the high-involvement vulnerable portrayal group compared with both the vulnerable portrayal group (*t*_433_=1.75; *P*=.08; *d*=0.21) and the control group (*t*_433_=1.89; *P*=.06; *d*=0.22), supporting hypotheses 8e and 9e. No significant difference in trust was observed between the vulnerable portrayal group and the control group. These findings suggest that increased vulnerability through increased involvement in the portrayal of physicians positively influences trust to a certain extent.

In addition, regression models were used, with model 4 and the control group serving as the reference (Multimedia Appendix 1). Although the direct impact of a simple portrayal of vulnerable physicians on evaluations was not significant (*b*=0.07; *P*=.17), the incorporation of content aimed at increasing involvement had a positive effect on medical professional evaluations (*b*=0.14; *P*=.006), which further positively influenced trust in physicians (*b*=0.93; *P*<.001); the indirect effect was 0.13 (95% CI 0.02-0.26).

### Discussion

Study 4’s questionnaire experiment showed that emphasizing medical care’s relevance in news articles can increase situational involvement for those with low to moderate enduring involvement, positively affecting their perceptions of vulnerable physician portrayals. This approach broadens the research’s applicability. Among the medical stereotype dimensions, physicians’ warmth was the most affected, which aligns with previous findings, whereas competence evaluations remained stable, highlighting the stronger impact of vulnerability portrayals on warmth and morality. Future media portrayals focusing on these aspects may increase positive audience responses.

## General Discussion

### Principal Findings

The 4 studies collectively examined the impact of vulnerable physician portrayals on medical professional evaluations considering varying individual involvement levels. Importantly, the participants’ reported media exposure supports the relevance of our focus—in study 1, a total of 62.6% (311/497) of respondents indicated moderate or higher regular exposure to vulnerable physician portrayals (mean 3.03, SD 0.78), and in studies 2 to 4, the participants’ average exposure to malicious or negative physician-related news ranged from 2.66 to 3.34 on a 5-point scale. These results suggest that such media content is commonly encountered, supporting the ecological validity of our focus.

Specifically, studies 1 and 2 revealed that, for individuals with low (infrequent hospital visits) and moderate (medium frequency of hospital visits) enduring involvement, vulnerable physician portrayals positively influenced medical professional evaluations. This positive effect was strongest among those with moderate enduring involvement, for whom the vulnerable portrayals indirectly increased trust in physicians through improved evaluations. However, for individuals with high involvement levels who frequently visited hospitals, the positive impact of vulnerable physician portrayals on evaluations diminished and became nonsignificant. Study 2 further revealed that, for individuals currently in a hospital setting, vulnerable physician portrayals could even result in negative effects. Therefore, caution should be taken when pushing such portrayals in spaces frequented by highly involved individuals—such as hospital-affiliated platforms or patient-centered online communities—where unintended negative reactions may occur.

Overall, studies 1 and 2 revealed that, for individuals with low to moderate enduring involvement (ie, those who almost never, seldom, or moderately visit hospitals), increasing involvement levels amplified the positive effects of vulnerable physician portrayals. Notably, this demographic represented most of the nonpatient population in our sample (378/492, 76.8% to 339/366, 92.6%). To maximize the positive impact of vulnerable physician media portrayals on this majority group considering the difficulty of manipulating enduring involvement, studies 3 and 4 explored whether situational involvement could be adjusted to further increase the positive effects of these portrayals. Studies 3a and 3b primarily aimed to provide causal evidence for the psychological mechanisms linking situational involvement to media persuasion and found that imagining unrelated others in medical situations (low situational involvement) could diminish the positive influence of these portrayals, whereas imagining oneself seeking treatment (high situational involvement) increased the positive effect. Building on these findings, study 4 tested a more ecologically valid and scalable manipulation by embedding situational involvement cues (eg, “medical issues concern everyone”) within the article itself, which significantly boosted the positive impact of vulnerable physician portrayals on public opinion and trust in physicians.

Furthermore, all 4 studies confirmed that the 3D stereotype content model fit better than the 2D model (Multimedia Appendix 1). Warmth was consistently influenced by the interaction between vulnerable portrayals and involvement levels across all studies, indicating enduring sensitivity to this dimension. Morality was also significantly affected by interaction effects in studies 1, 3b, and 4, with effect sizes ranking highest in studies 1 and 3b. Finally, competence had no significant interaction effects; only in the moderate-involvement groups in studies 3a and 3b did the vulnerable portrayal group rate physicians’ competence significantly higher than the control group. This result suggests that public evaluations of competence are less influenced by vulnerable physician portrayals, aligning with findings that patients prioritize benevolence over competence [33,34]. This finding may be due to the cultural belief in physicians’ inherent competence [42], indicating that altering competence evaluations may require more extensive interventions beyond singular news exposure [43].

### Study Significance

This study explores the nuanced effects of vulnerable physician portrayals on individuals with varying involvement levels, filling a gap in the research largely focused on negative or positive portrayals of physicians [44,45]. By examining the impact of neutral, vulnerable physician portrayals, this research enhances our understanding of how such portrayals influence physician-patient trust and supports the 3D model of stereotype content, affirming the independence of the morality and warmth dimensions.

In addition, unlike marginalized groups typically depicted in vulnerable media portrayals [46,47], physicians generally hold a higher social standing. The effects observed in this study might also extend to other professions, such as teachers and civil servants.

Practically, these findings have significant implications for public trust in health care systems, particularly during public health crises such as the COVID-19 pandemic. Studies have shown that media portrayals of health care workers’ vulnerabilities, such as during COVID-19 vaccination campaigns, can significantly influence public trust [48,49]. The observed inverted U–shaped relationship in this study suggests that, while some vulnerability can humanize health care workers and foster empathy, an excessive focus on it could erode trust.

On the basis of these results, this study offers the following practical recommendations. First, for audiences involving patients, such as those in hospitals or online health platforms, exposure to vulnerability-themed portrayals (eg, physicians shown as exhausted or underpaid) may evoke negative evaluations. Medical institutions and media outlets should avoid pushing or displaying such content within physical hospital settings or patient apps to prevent unintended negative perceptions. Second, when vulnerable physician portrayals are presented alongside unrelated or highly emotional health appeals, such as fundraising campaigns, this may lower reader engagement with the physician portrayal and amplify defensive or negative reactions. Editorial discretion is advised when curating health-related content bundles. Third, for the public, who typically exhibits low to moderate involvement levels, highlighting the relevance of health issues (eg, “Everyone is closely related to seeking medical treatment”) can enhance situational involvement and strengthen the positive impact of vulnerable portrayals. Fourth, across the studies, perceptions of physician competence remained stable, whereas warmth and morality were more malleable and positively influenced by moderate involvement. Thus, media narratives aimed at building trust may benefit from emphasizing empathy, integrity, and relational care rather than professional hardship alone.

### Limitations and Future Directions

This study relied on self-reports for explicit stereotypes, which may be influenced by personal biases and societal norms [50]. Moreover, the evaluation scale included only 2 items per dimension (competence, morality, and warmth), which may have affected internal consistency. Future research could improve reliability by using longer, well-validated scales and should investigate implicit stereotypes for a more comprehensive understanding.

This study also grouped 3 types of vulnerable physician portrayals—those involving overwork, low compensation, and violence exposure—without examining their distinct effects. Although this approach enhanced ecological validity and reflected common media narratives, it may have masked meaningful differences. For example, portrayals of injured physicians may impact trust, professional identity, or physician-patient expectations differently than portrayals of fatigue or burnout [51]. Future studies should explore these subtypes separately to clarify how specific forms of vulnerability influence public perceptions and communication dynamics in health care.

The experimental design focused on immediate effects, with study 1 using cross-sectional data that do not capture long-term outcomes. Longitudinal studies are needed to assess these effects over time and establish the causal relationship between stereotypes and trust.

Another limitation is the difference in impact between static portrayals and video portrayals, such as those in television dramas or streaming media, which often depict health care professionals in exaggerated ways [52,53]. Future research should consider these media formats.

In addition, the studies’ findings are limited by the sample’s focus on Chinese participants, which may affect generalizability. While media portrayals of physicians as vulnerable professionals are common worldwide—in countries such as India, the United States, and Australia—cultural differences in trust, professional norms, and health care systems may influence how such portrayals are interpreted. The observed effects, including the inverted U–shaped role of involvement, may not manifest similarly across contexts. Nonetheless, the framework developed in this study offers a useful foundation for understanding how emotionally ambiguous health-related content influences public perceptions across media systems. Future research should adopt cross-cultural or comparative designs to test the robustness of these mechanisms and inform international strategies for health communication, media ethics, and physician-patient trust building.

Moreover, this study contributes to the media and communication research by suggesting that public responses to vulnerable physician portrayals may extend beyond evaluations of physicians to perceptions of the media channels delivering such messages. Emotional responses to content can influence how audiences perceive the source and their engagement [54,55]. Thus, when media platforms successfully evoke empathy through vulnerable physician portrayals, they may enhance their perceived trustworthiness. Conversely, if such portrayals are perceived as manipulative or overly negative, they may undermine trust in the media outlet. While our study focused primarily on the audience’s evaluation of physicians, future research should consider the use of qualitative or mixed methods to explore how audiences articulate their emotional reactions to both the portrayals and the channels disseminating them. Such an approach could deepen our understanding of the media-audience-profession dynamic and offer practical guidance for responsible health communication.

Finally, highly involved patients yielded lower evaluations after they viewed vulnerable physician portrayals, highlighting the need for careful management of such content. Future research should explore whether positive portrayals can improve perceptions among these patients.

### Conclusions

Exposure to neutral vulnerable physician portrayals is positively correlated with trust in physicians, suggesting a long-term positive impact on the sociomedical environment. However, this impact varies by involvement level. For individuals with low to moderate hospital visit frequencies (ie, involvement), increased involvement enhances the positive effect of vulnerable physician portrayals on physician evaluations and trust. In contrast, those with high visit frequencies exhibit no effect, and the superhighly involved population who are currently receiving medical treatment may even experience negative effects. This finding indicates an inverted U–shaped moderation effect of involvement on the impact of vulnerable physician portrayals.

In the general population, nearly 80% of individuals have low or moderate hospital visit frequencies. For these individuals, emphasizing the relevance of “medical matters concern everyone” could increase their involvement level and, thus, the effectiveness of exposure to vulnerable physician media portrayals.

## Appendix

Multimedia Appendix 1 Essential experimental materials, including the questionnaire used in the study and additional results and analyses.

## Abbreviations

ELM: elaboration likelihood model

## Data Availability

The data that support the findings of this study and the information needed to reproduce all the reported methodologies are openly available in the Open Science Framework [56].
