# Supplementary material for: Impact of News Portrayals of Physicians as Vulnerable on the Public’s Evaluation and Trust in Physicians Under Different Involvement Levels: Quantitative Study
Source: J Med Internet Res. 2025 Jul 9;27:e67947. doi: 10.2196/67947 (PMC12287668; doi:10.2196/67947)
Supplement: Multimedia Appendix 1 [file jmir_v27i1e67947_app1.docx]

# Online Supplementary Material

### Section 1: Materials of Studies

#### Section 1a. Questionnaire of Study 1

您好！非常感谢您的参加。本调查问卷将匿名进行，调查问卷搜集的所有信息将仅供课题研究使用，未经您的同意，不会用于其他用途。请您根据自己的真实情况回答问卷中的问题，答案没有对错之分。

**【持续性卷入度】**

您近两年内去医院的频率？1=基本没有，5=非常多 ○1 ○2 ○3 ○4 ○5

**【日常伤医浏览】**

请问您过去两年内看到以下类型报道的频率如何？1=几乎没有，5=非常多

1. 有关恶性伤医、医闹类事件的报道，如《突发迟到伤医时间，江苏三甲医生被刺》
2. 体现医生工作辛苦、压力大等工作困境的报道，如《医生一周工作120小时，过度劳累苦不堪言》
3. 体现医生待遇糟糕，发展受限等职业困境的报道，如《我国基层医生待遇低，过半人认为职业前景一般》

**【注意检查题】**

您是否每晚睡眠时间都小于1小时？ ○是 ○否

**【刻板印象】**

请您根据自己对医生群体的看法，选择对以下观点的赞同度。1=完全不同意，5=非常同意

1. 医生们是待人热情的 ○1 ○2 ○3 ○4 ○5
2. 医生们是友好亲和的 ○1 ○2 ○3 ○4 ○5
3. 医生们是有能力的 ○1 ○2 ○3 ○4 ○5
4. 医生们是有才华的 ○1 ○2 ○3 ○4 ○5
5. 医生们是值得信赖的 ○1 ○2 ○3 ○4 ○5
6. 医生们是诚实正直的 ○1 ○2 ○3 ○4 ○5

**【对医信任】**

请您根据自己对医生群体的看法，选择对以下观点的赞同度。1=完全不同意，5=非常同意

1. 我觉得医生是真的关心患者。 ○1 ○2 ○3 ○4 ○5
2. 我相信医生对所有的患者都是一视同仁的。 ○1 ○2 ○3 ○4 ○5
3. 我觉得医生即使有时间， 也不会与患者耐心沟通。（反）○1 ○2 ○3 ○4 ○5
4. 医生能够为患者的治疗尽心尽力。 ○1 ○2 ○3 ○4 ○5
5. 总的来说，我信任医生。 ○1 ○2 ○3 ○4 ○5
6. 医生的医术没有他们应该有的那样好。（反）○1 ○2 ○3 ○4 ○5

**【人口学信息】**

您的性别：○男 ○女

您的年龄：______

您的学历：○小学及以下 ○初中 ○高中 ○中专 ○大专 ○本科 ○硕士 ○博士

您的家庭平均月收入大概是：○2500元以下 ○2501-6500元 ○6501-12500元 ○12500-25000元 ○25000-80000元 ○80000元以上

您目前身体状况：○很差 ○较差 ○一般 ○较好 ○很好

###### English version

Hello! We sincerely appreciate your participation. This survey will be conducted anonymously, and all information collected from the survey will be used solely for research purposes. Without your consent, it will not be used for any other purposes. Please answer the questions in the questionnaire truthfully, as there are no right or wrong answers.

**[Enduring Involvement]**

How often have you visited hospitals in the past two years?

1 = Almost never, 2 = Rarely, 3 = Occasionally, 4 = Frequently, 5 = Very frequently

**[****Exposure to News Portraying Vulnerable Doctors.]**

How often have you come across the following types of news reports in the past two years? 1 = Almost never, 5 = Very frequently

1. Reports about malicious violence against doctors or medical disputes, such as "Doctor Stabbed Due to Delay in Arrival, Jiangsu Top-Tier Hospital Incident"
2. Reports highlighting the hardships and pressures faced by doctors in their work, such as "Doctor Works 120 Hours a Week, Overworked and Miserable"
3. Reports highlighting poor compensation and limited career prospects for doctors, such as "Low Compensation for Grassroots Doctors, More Than Half Feel Moderate Career Prospects"

**[Attention Check]**

Do you sleep for less than 1 hour every night? ○ Yes ○ No

**[Stereotypical Impressions]**

Please rate your agreement level based on your perception of the physician group for the following statements. 1 = Completely disagree, 5 = Strongly agree

1. Doctors are warm in their interactions with people.
2. Doctors are friendly and approachable.
3. Doctors are competent in their profession.
4. Doctors are talented individuals.
5. Doctors are trustworthy.
6. Doctors are honest and upright individuals.

**[Trust in Doctors]**

Please rate your agreement level based on your perception of the physician group for the following statements. 1 = Completely disagree, 5 = Strongly agree

1. I believe doctors genuinely care about their patients.
2. I trust that doctors treat all patients equally.
3. Even when they have time, I don't think doctors are patient in communicating with patients. (Reversed)
4. Doctors put their heart and soul into treating patients.
5. Overall, I trust doctors.
6. Doctors' medical skills are not as good as they should be. (Reversed)

**[Demographic Information]**

Your gender: ○ Male ○ Female

Your age: ______

Your education level: ○ Elementary school or below ○ Middle school ○ High school ○ Vocational school ○ College ○ Bachelor's degree ○ Master's degree ○ Doctorate

Your family's average monthly income is approximately:○Below 2500 ○2501-6500 ○6501-12500 ○12501-25000 ○25001-80000 ○Above 80000

Your current physical condition: ○ Very poor ○ Poor ○ Average ○ Good ○ Very good

#### Section 1b. Experimental Procedure of Study 3b

**Step 1. participants were randomly assigned to the following three scenarios:**

**情景1(中卷入组)：**近两个月来，**你**反复发烧。**你**吃过抗生素消炎来尝试排除感染，但今天**你**又发烧了。于是**你**来到了一家综合门诊，医生询问完基本症状后，要求做完血常规检查再来问诊。于是**你**开始排队做血常规。

请代入地想象并描述一下你此时会有怎样的想法或情绪？不少于三个词

***English version-****Situation 1(moderate involvement):* ***You****'ve had a recurring fever for the last two months.* ***You*** *took antibiotics to get rid of the infection, but the fever came back today. So* ***you*** *go to a general clinic. The doctor asks you about your basic symptoms and then asks you to do a blood test before coming back. So* ***you*** *start lining up for the blood test.*

Please imagine and describe your thoughts or emotions at this moment. Use a minimum of three words.

**情境2(低卷入组)：**近两个月来，**王先生**反复发烧。**他**吃过抗生素消炎来尝试排除感染，但今天又发烧了。于是**王先生**来到了一家综合门诊，医生询问完基本症状后，要求做完血常规检查再来问诊。于是**王先生**开始排队做血常规。

请简单描述一下今天的天气，不少于三个词

***English version-****Situation 2(low involvement):* ***Mr. Wang*** *has had a recurring fever for the last two months.* ***He*** *took antibiotics trying to get rid of the infection, but the fever came back today. So* ***Mr. Wang*** *goes to a general clinic. The doctor asks him about his basic symptoms and then asks him to do a blood test before coming back. So* ***Mr. Wang*** *began to line up for the blood test.*

Please provide a brief description of today's weather in a minimum of three words.

**情境3（无卷入组）：**无(跳过这一阅读想象步骤)

***English version-****Situation 3(none involvement): Noting.****[Skip the imagery step]***

**Step 2. Stereotyping was measured for the first time**

请您根据自己对医生群体的看法，选择对以下观点的赞同度。1=完全不同意，5=非常同意

1. 医生们是待人热情的 ○1 ○2 ○3 ○4 ○5

2. 医生们是友好亲和的 ○1 ○2 ○3 ○4 ○5

3. 医生们是有能力的 ○1 ○2 ○3 ○4 ○5

4. 医生们是有才华的 ○1 ○2 ○3 ○4 ○5

5. 医生们是值得信赖的 ○1 ○2 ○3 ○4 ○5

6. 医生们是诚实正直的 ○1 ○2 ○3 ○4 ○5

***English version-***Please indicate your level of agreement with the following statements based on your perception of the medical profession. Rate each statement on a scale of 1 to 5, where 1 indicates 'strongly disagree' and 5 indicates 'strongly agree'.

1 Doctors are warm and welcoming. ○1 ○2 ○3 ○4 ○5

2 Doctors are friendly and approachable. ○1 ○2 ○3 ○4 ○5

3 Doctors are competent. ○1 ○2 ○3 ○4 ○5

4 Doctors are talented. ○1 ○2 ○3 ○4 ○5

5 Doctors are trustworthy. ○1 ○2 ○3 ○4 ○5

6 Doctors are honest and upright. ○1 ○2 ○3 ○4 ○5

**Step 3.** **The participants were redirected to finish an unrelated 12-minute cognitive task.**

**Step 4. Participants were randomly assigned to read the following two materials.**

While waiting for the results, you/Mr. Wang came across the following news :

*[The news of vulnerable portrayals]**-Studies 2, 3a, 3b vulnerable materials were the same*


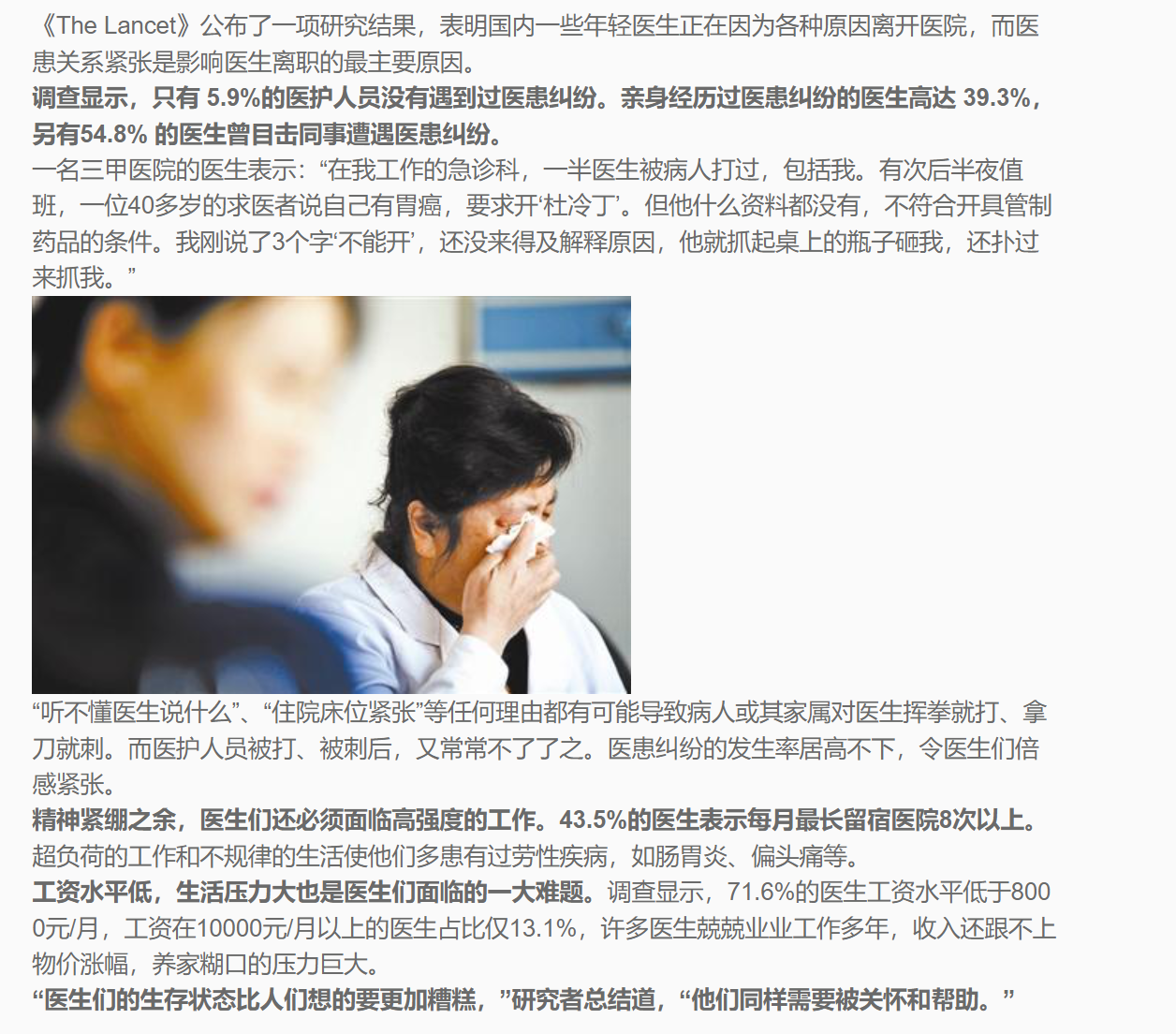


*[The news of vulnerable portrayals]-****English version:***

《The Lancet》has published a study showing that some young doctors in China are leaving hospitals for various reasons, with tense doctor-patient relationships being the main cause of their resignations.

**The survey revealed that only 5.9% of medical staff have never encountered a doctor-patient dispute. A staggering 39.3% of doctors have personally experienced disputes, and 54.8% have witnessed their colleagues dealing with such conflicts.**

A doctor from a top-tier hospital said, "In the emergency department where I work, half of the doctors, including myself, have been hit by patients. Once during a night shift, a man in his 40s claimed he had stomach cancer and demanded a prescription for 'Duludin'. He didn't have any documentation, which was required for prescribing controlled drugs. I had barely said three words—'I can't prescribe'—before I could explain the reason, he grabbed a bottle from the table and smashed it at me and tried to grab me."

[Figure]

Any reason, such as "not understanding what the doctor is saying" or "a shortage of hospital beds," can lead to patients or their families hitting or even stabbing doctors. After such incidents, medical staff are often left without recourse. The high occurrence of doctor-patient disputes leaves doctors feeling increasingly tense.

**In addition to the mental strain, doctors also face high-intensity work. 43.5% of the doctors said they used to put up for the night at the hospitals more than 8 times per month.** Overloaded work and irregular lifestyles lead to them suffering from fatigue-related diseases, such as gastroenteritis and migraine.

**Low salaries and high living pressures are also major challenges for doctors.** The survey showed that 71.6% of doctors earn less than 8,000 RMB per month, and only 13.1% earn more than 10,000 RMB per month. Many doctors have worked diligently for years, but their incomes still don't keep up with the rising cost of living, resulting in enormous pressure to support their families.

**"Doctors’ living conditions are even worse than people think," the researchers concluded. "They also need care and help."**

*[The news of control groups]-Studies 2, 3a,3b,4 control materials were the same*


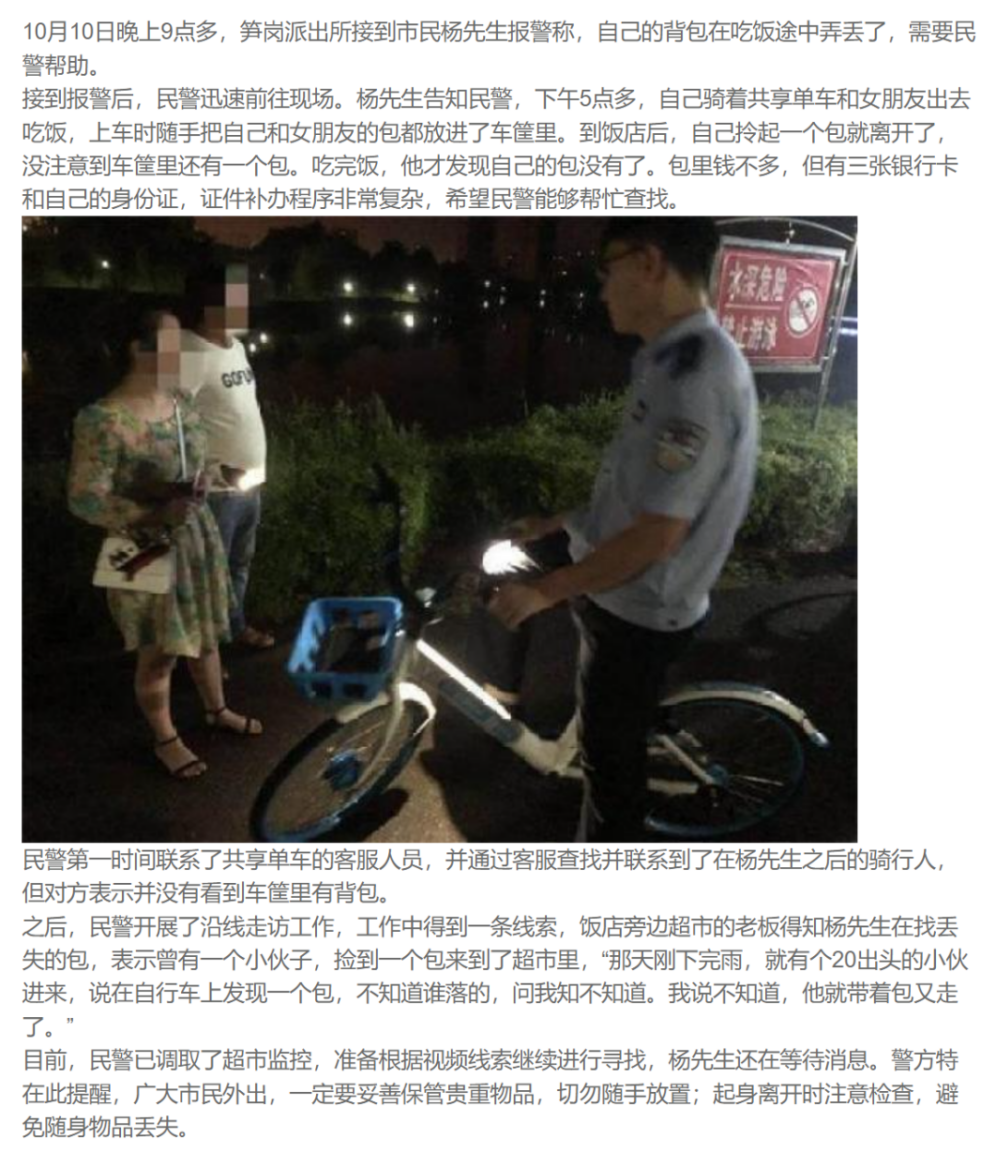


*[The news of control groups]-****English version:***

On the evening of October 10th, at around 9 PM, Mr. Yang, a citizen, reported to the Sungang Police Station that he had lost his backpack while dining out and needed police assistance.

Upon receiving the report, the police quickly went to the scene. Mr. Yang informed the police that at around 5 PM, he rode a shared bicycle with his girlfriend to go out for dinner. When they got on the bike, he casually placed both his and his girlfriend's bags in the bike's basket. When they arrived at the restaurant, he picked up one bag and left, not noticing that there was another bag in the basket. After finishing their meal, he realized that his bag was gone. There wasn't much money in the bag, but there were three bank cards and his ID card, and the process of reissuing the documents was very complicated, so he hoped the police could help find the bag.

[Figure]

The police immediately contacted the customer service staff of the shared bicycle company and, through them, found and contacted the rider who used the bike after Mr. Yang. However, the rider said that they did not see a backpack in the basket.

Subsequently, the police conducted a visit along the route and got a clue during their investigation. The owner of a supermarket next to the restaurant learned that Mr. Yang was looking for his lost bag and said that a young man had picked up a bag and brought it to the supermarket. "It had just stopped raining that day when a young man in his early 20s came in, saying that he found a bag on a bike and didn't know whose it was. He asked if I knew. I said I didn't know, so he took the bag and left."

At present, the police have obtained the supermarket's surveillance footage and are preparing to continue the search based on the video clues. Mr. Yang is still waiting for news. The police hereby remind the public to properly secure their valuables when going out and not to place them casually. When leaving, check your belongings to avoid losing personal items.

**Step 5. Stereotyping was measured for the second time**

请您根据自己对医生群体的看法，选择对以下观点的赞同度。1=完全不同意，5=非常同意

1. 医生们是待人热情的 ○1 ○2 ○3 ○4 ○5

2. 医生们是友好亲和的 ○1 ○2 ○3 ○4 ○5

3. 医生们是有能力的 ○1 ○2 ○3 ○4 ○5

4. 医生们是有才华的 ○1 ○2 ○3 ○4 ○5

5. 医生们是值得信赖的 ○1 ○2 ○3 ○4 ○5

6. 医生们是诚实正直的 ○1 ○2 ○3 ○4 ○5

***English version-***Please indicate your level of agreement with the following statements based on your perception of the medical profession. Rate each statement on a scale of 1 to 5, where 1 indicates 'strongly disagree' and 5 indicates 'strongly agree'.

1 Doctors are warm and welcoming. ○1 ○2 ○3 ○4 ○5

2 Doctors are friendly and approachable. ○1 ○2 ○3 ○4 ○5

3 Doctors are competent. ○1 ○2 ○3 ○4 ○5

4 Doctors are talented. ○1 ○2 ○3 ○4 ○5

5 Doctors are trustworthy. ○1 ○2 ○3 ○4 ○5

6 Doctors are honest and upright. ○1 ○2 ○3 ○4 ○5

**Step 6. The participants were redirected to finish an unrelated 12-minute cognitive task again.**

**Step 7. Participants were asked to read the comics corresponding to their respective groups and then their trust in doctors was measured.**

Finally, you/Mr. Wang received your complete blood count (CBC) test results and had the following conversation with the doctor:

*Situation 1(moderate involvement):*


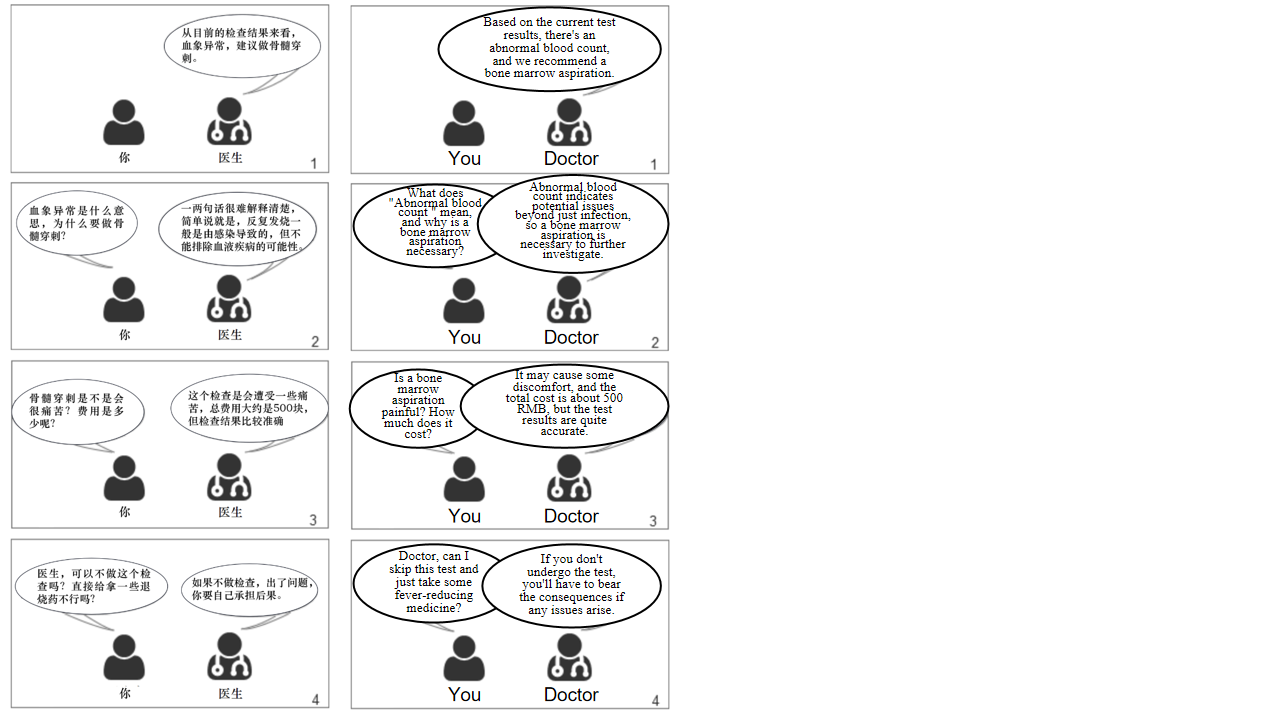


*Situation 2or3(low or no involvement):*


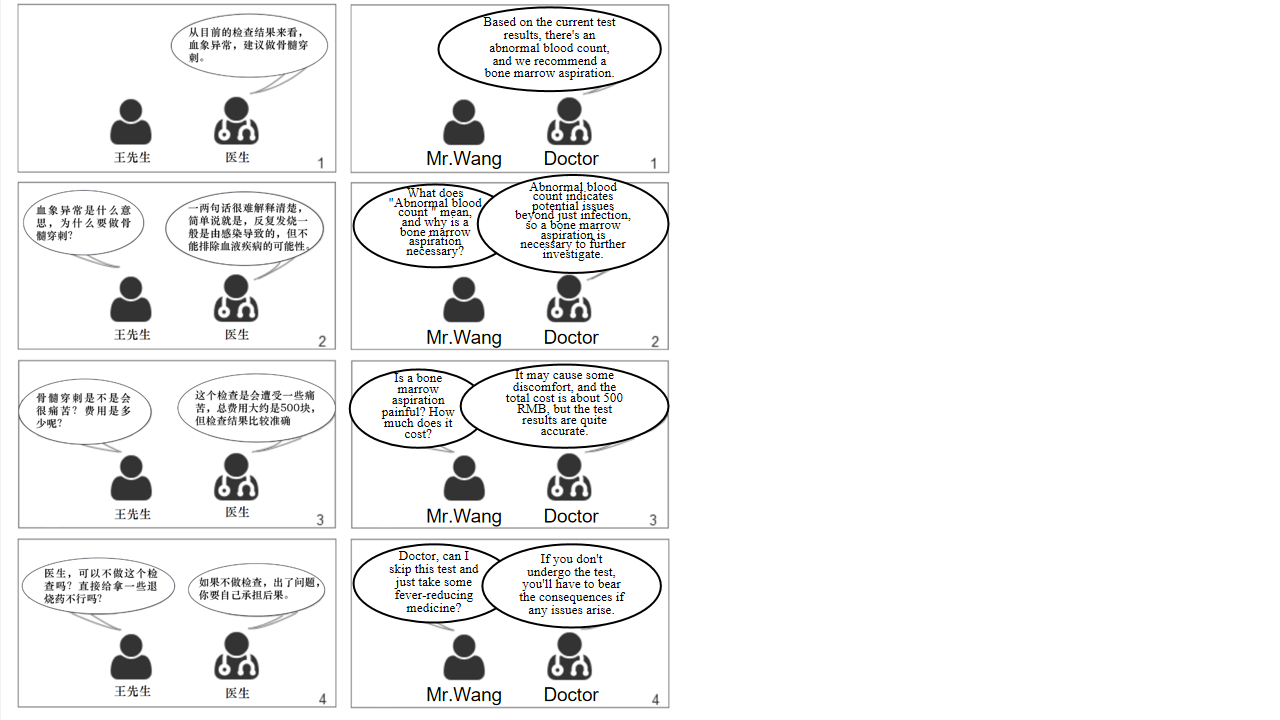


**【注意检验题1】**该项目的费用是：○200 ○500 ○1000

**【对医信任】**

请你根据自己想法对以下说法进行评分，1=非常不同意，5=非常同意

1. 医生能够及时询问患者的病情 ○1 ○2 ○3 ○4 ○5
2. 我觉得医生是真的关心患者 ○1 ○2 ○3 ○4 ○5
3. 医生的治疗效果比我预想的要好 ○1 ○2 ○3 ○4 ○5
4. 这所医院的流程是高效的 ○1 ○2 ○3 ○4 ○5
5. 我相信医生对所有的患者都是一视同仁的 ○1 ○2 ○3 ○4 ○5
6. 医生能够及时回答我的疑问 ○1 ○2 ○3 ○4 ○5
7. 我以后还会找这位医生看病 ○1 ○2 ○3 ○4 ○5
8. 我觉得医生即使有时间，也不会与我耐心沟通（反） ○1 ○2 ○3 ○4 ○5
9. 医生的治疗过程跟我想的差不多 ○1 ○2 ○3 ○4 ○5
10. 我会介绍我的朋友、家人找这位医生看病 ○1 ○2 ○3 ○4 ○5
11. 我相信医生会在我需要时为我提供个性化服务 ○1 ○2 ○3 ○4 ○5
12. 我对为患者治疗的医生还是满意的 ○1 ○2 ○3 ○4 ○5
13. 医生能够为患者的治疗尽心尽力 ○1 ○2 ○3 ○4 ○5

***English version-*[Attention Check]**The cost for this item is: ○200 ○500 ○1000

**[Trust in the Doctor]**

Please rate the following statements based on your thoughts, 1=strongly disagree, 5=strongly agree:

1. Doctors can promptly inquire about the patient's condition. ○1 ○2 ○3 ○4 ○5
2. I believe doctors genuinely care about the patients. ○1 ○2 ○3 ○4 ○5
3. The treatment effectiveness from the doctor is better than I expected. ○1 ○2 ○3 ○4 ○5
4. The hospital's procedures are efficient. ○1 ○2 ○3 ○4 ○5
5. I trust that doctors treat all patients equally. ○1 ○2 ○3 ○4 ○5
6. Doctors can promptly answer my questions. ○1 ○2 ○3 ○4 ○5
7. I would seek treatment from this doctor in the future. ○1 ○2 ○3 ○4 ○5
8. I believe that even if the doctor has time, they won't communicate patiently with me (reverse). ○1 ○2 ○3 ○4 ○5
9. The doctor's treatment process is similar to what I expected. ○1 ○2 ○3 ○4 ○5
10. I would recommend this doctor to my friends and family for treatment. ○1 ○2 ○3 ○4 ○5
11. I trust that the doctor will provide personalized service when I need it. ○1 ○2 ○3 ○4 ○5
12. Overall, I am satisfied with the doctors treating patients. ○1 ○2 ○3 ○4 ○5
13. Doctors make every effort for the patient's treatment. ○1 ○2 ○3 ○4 ○5

**Step 8. Participants were asked to provide demographic information.**

您的性别：○男 ○女

您的年龄：______

您的学历：○小学及以下 ○初中 ○高中 ○中专 ○大专 ○本科 ○硕士 ○博士

***English version-***Gender: ○ Male ○ Female.

Age: ______.

Education level: ○ Primary school or below ○ Junior high school ○ High school ○ Vocational school ○ College ○ Master's degree ○ Doctoral degree.

#### Section 1c. Three sets of reading materials for Study 4

*[The news of vulnerable portrayals with high involvement]*


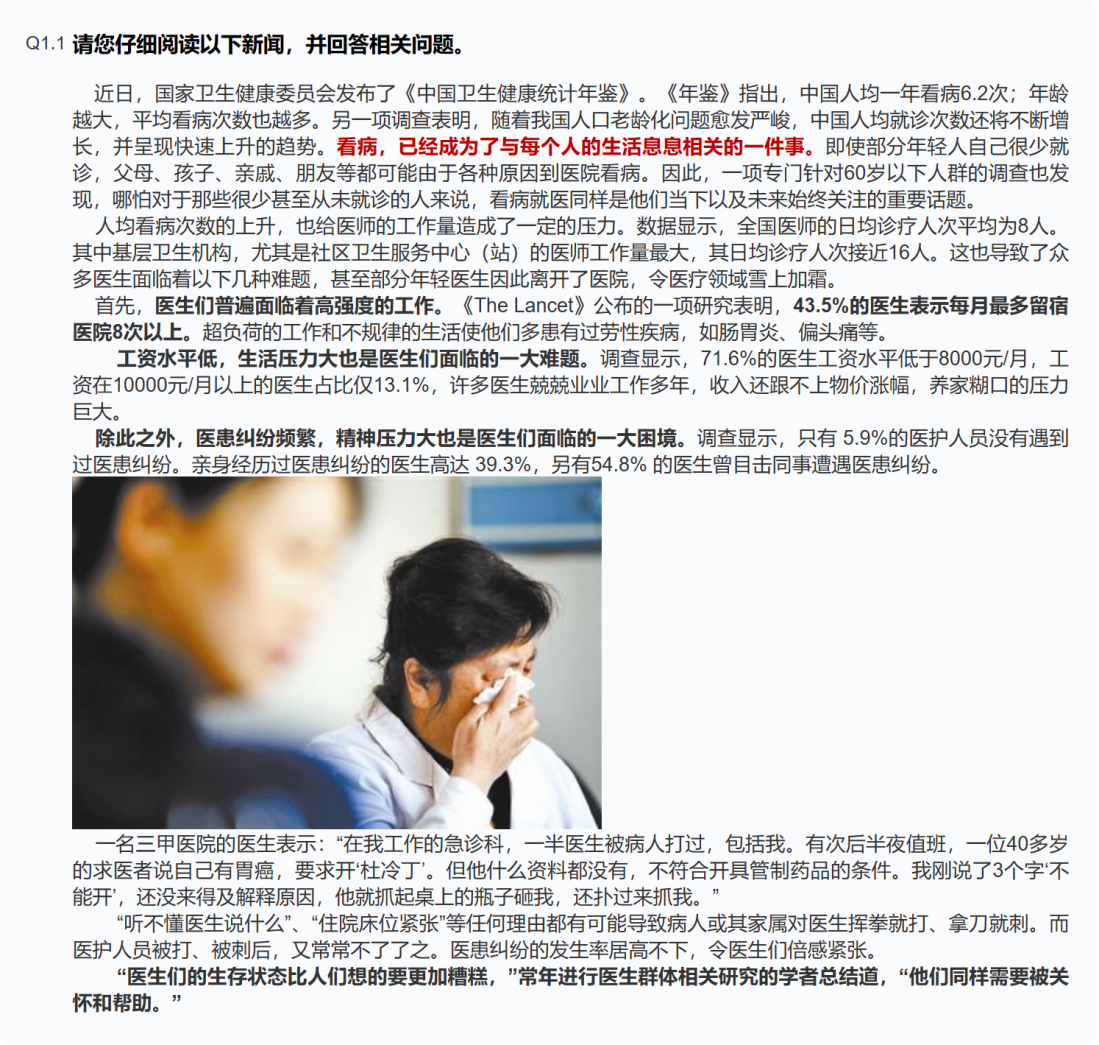


*[The news of vulnerable portrayals with high involvement]**-****English version:***

Recently, the National Health Commission of China released the "China Health and Health Statistics Yearbook." The "Yearbook" points out that the average number of doctor visits per year for Chinese citizens is 6.2 times; as age increases, the average number of doctor visits also increases. Another survey indicates that with the increasingly severe problem of China's aging population, the average number of medical visits per person will continue to rise, showing a rapid upward trend. **Seeking medical treatment has become an integral part of everyone's life.** Even for young people who seldom seek medical treatment themselves, parents, children, relatives, friends, and others may visit hospitals for various reasons. Therefore, a survey specifically targeting individuals under the age of 60 also revealed that even for those who rarely or never seek medical treatment, healthcare remains a significant and continuous concern for them.

The rising average number of doctor visits per person also exerts certain pressure on healthcare professionals. Data reveals that the daily average number of patients treated by physicians nationwide is 8. Among them, physicians in grassroots healthcare institutions, particularly in community health service centers, manage the highest patient workload, with an average of nearly 16 patients treated per day. This situation presents numerous challenges to doctors, and some young physicians have even left hospitals due to these challenges, further exacerbating the already burdensome healthcare sector.

**Firstly, doctors commonly face high-intensity work.** A study published in "The Lancet" indicates that 43.5% of doctors used to put up for the night at the hospitals more than 8 times per month. Overloaded work and irregular lifestyles lead to them suffering from fatigue-related diseases, such as gastroenteritis and migraine.

**Low salaries and high living pressures are also major challenges for doctors.** The survey showed that 71.6% of doctors earn less than 8,000 RMB per month, and only 13.1% earn more than 10,000 RMB per month. Many doctors have worked diligently for years, but their incomes still don't keep up with the rising cost of living, resulting in enormous pressure to support their families.

**Furthermore, frequent doctor-patient disputes and significant mental stress form another major predicament for healthcare professionals**. The survey reveals that only 5.9% of medical staff have never encountered a doctor-patient dispute. A staggering 39.3% of doctors have personally experienced disputes, and 54.8% have witnessed their colleagues dealing with such conflicts.

[figure]

A doctor from a top-tier hospital said, "In the emergency department where I work, half of the doctors, including myself, have been hit by patients. Once during a night shift, a man in his 40s claimed he had stomach cancer and demanded a prescription for 'Duludin'. He didn't have any documentation, which was required for prescribing controlled drugs. I had barely said three words—'I can't prescribe'—before I could explain the reason, he grabbed a bottle from the table and smashed it at me and tried to grab me."

Any reason, such as "not understanding what the doctor is saying" or "a shortage of hospital beds," can lead to patients or their families hitting or even stabbing doctors. After such incidents, medical staff are often left without recourse. The high occurrence of doctor-patient disputes leaves doctors feeling increasingly tense.

**"Doctors’ living conditions are even worse than people think," scholars who consistently study the medical profession concluded, "They also need care and help."**

*[The news of vulnerable portrayals]*

*
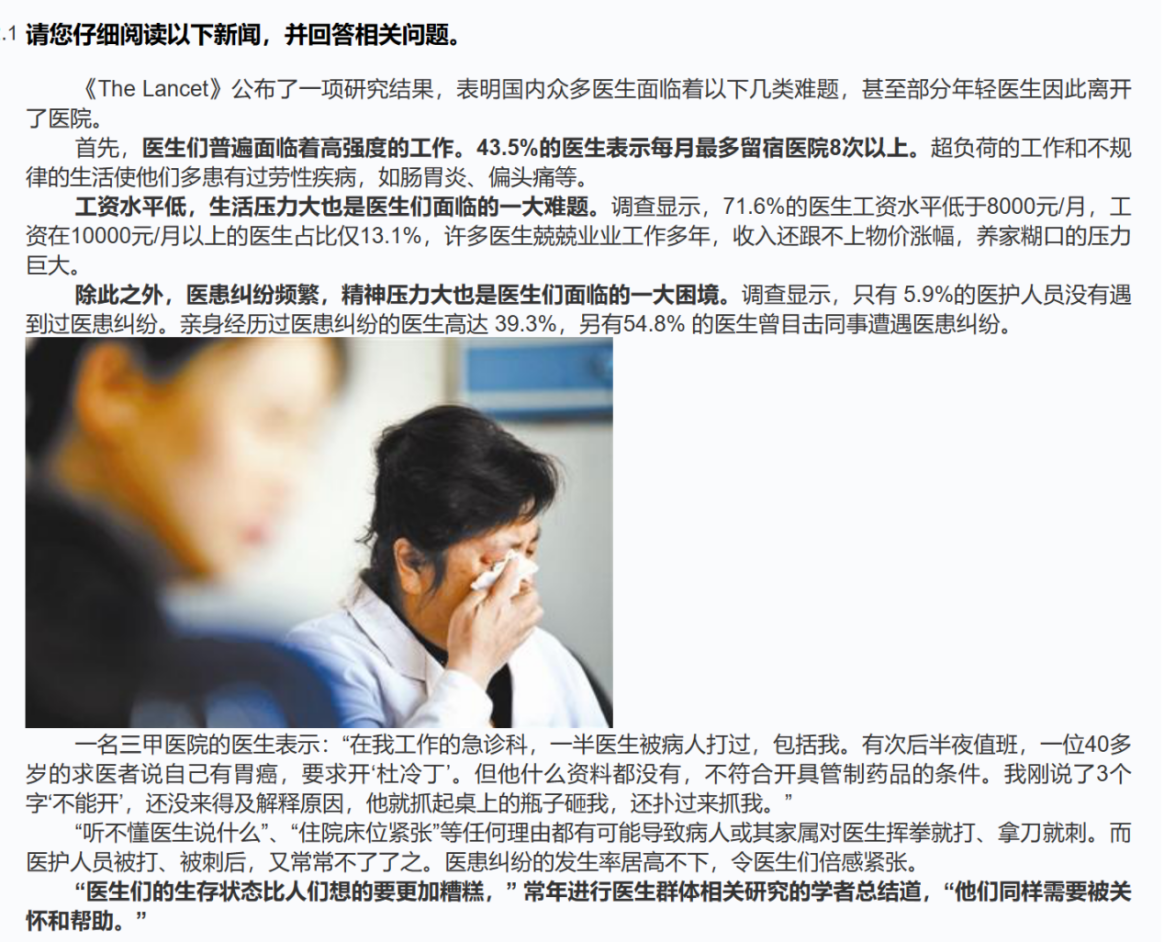
*

*[The news of vulnerable portrayals]-****English version:***

《The Lancet》has released a research study revealing that numerous doctors within the country are confronting various challenges, and some young physicians have even departed from hospitals as a result.

**Firstly, doctors commonly face high-intensity work.** **As many as 43.5% of doctors used to put up for the night at the hospitals more than 8 times per month.** Overloaded work and irregular lifestyles lead to them suffering from fatigue-related diseases, such as gastroenteritis and migraine.

**Low salaries and high living pressures are also major challenges for doctors.** The survey showed that 71.6% of doctors earn less than 8,000 RMB per month, and only 13.1% earn more than 10,000 RMB per month. Many doctors have worked diligently for years, but their incomes still don't keep up with the rising cost of living, resulting in enormous pressure to support their families.

**Furthermore, frequent doctor-patient disputes and significant mental stress form another major predicament for healthcare professionals**. The survey reveals that only 5.9% of medical staff have never encountered a doctor-patient dispute. A staggering 39.3% of doctors have personally experienced disputes, and 54.8% have witnessed their colleagues dealing with such conflicts.

[figure]

A doctor from a top-tier hospital said, "In the emergency department where I work, half of the doctors, including myself, have been hit by patients. Once during a night shift, a man in his 40s claimed he had stomach cancer and demanded a prescription for 'Duludin'. He didn't have any documentation, which was required for prescribing controlled drugs. I had barely said three words—'I can't prescribe'—before I could explain the reason, he grabbed a bottle from the table and smashed it at me and tried to grab me."

Any reason, such as "not understanding what the doctor is saying" or "a shortage of hospital beds," can lead to patients or their families hitting or even stabbing doctors. After such incidents, medical staff are often left without recourse. The high occurrence of doctor-patient disputes leaves doctors feeling increasingly tense.

**"Doctors’ living conditions are even worse than people think," scholars who consistently study the medical profession concluded, "They also need care and help."**

*[The news of Control Group] Same as Study 3b*

### Section 2: Supplemental Results for Study 1

#### Section 2a. Descriptive statistics and correlation in Study 1

|  | *M* | *SD* | 1 | 2 | 3 | 4 | 5 | 6 | 7 | 8 | 9 | 10 | 11 |
| --- | --- | --- | --- | --- | --- | --- | --- | --- | --- | --- | --- | --- | --- |
| 1 Sex | - | - |  |  |  |  |  |  |  |  |  |  |  |
| 2 Age | 31.72 | 8.64 | 0.15** |  |  |  |  |  |  |  |  |  |  |
| 3 Education Level | 5.83 | 1.16 | 0.10* | -.32*** |  |  |  |  |  |  |  |  |  |
| 4 Monthly Income | 3.53 | 1.02 | 0.11* | 0.19*** | 0.07 |  |  |  |  |  |  |  |  |
| 5 Health Status | 3.75 | 0.73 | 0.03 | -0.02 | 0.07 | -0.02 |  |  |  |  |  |  |  |
| 6 Exposure to Vulnerable Portrayals | 3.04 | 0.78 | -0.02 | -0.01 | 0.11* | 0.07 | 0.04 |  |  |  |  |  |  |
| 7 Enduring Involvement | 2.79 | 0.94 | -0.04 | 0.16*** | -0.11* | 0.23*** | -.23*** | 0.05 |  |  |  |  |  |
| 8 Warmth | 3.72 | 0.76 | 0.09* | 0.26*** | -0.21*** | 0.18*** | 0.08 | 0.15** | 0.22*** |  |  |  |  |
| 9 Competence | 4.32 | 0.59 | 0.06 | 0.12** | -0.15** | 0.11* | 0.00 | 0.07 | 0.15** | 0.49*** |  |  |  |
| 10 Morality | 4.09 | 0.63 | 0.02 | 0.16*** | -0.15** | 0.16*** | 0.07 | 0.12** | 0.17*** | 0.64*** | 0.57*** |  |  |
| 11 Overall Stereotypes | 4.05 | 0.56 | 0.07 | 0.22*** | -0.20*** | 0.18*** | 0.06 | 0.14** | 0.21*** | 0.87*** | 0.79*** | 0.87*** |  |
| 12 Trust in Doctors | 3.92 | 0.59 | 0.06 | 0.19*** | -0.14** | 0.19*** | 0.05 | 0.15** | 0.24*** | 0.72*** | 0.59*** | 0.76*** | 0.82*** |
| **Note：****p*＜0.05, ***p*＜0.01, ****p*＜0.001, the same applies hereinafter。 | | | | | | | | | | | | | |

#### Section 2b. The moderated mediating models of Study 1

|  | Model 1：Overall Stereotypes | | | Model 2：Trust in Doctors | | |
| --- | --- | --- | --- | --- | --- | --- |
|  | *B* | *se* | *p* | *B* | *se* | *p* |
| Sex | 0.09 | 0.05 | 0.094 | -0.02 | 0.03 | 0.611 |
| Age | 0.01 | 0.00 | 0.015 | 0.00 | 0.00 | 0.467 |
| Education Level | -0.09 | 0.02 | ＜0.001 | 0.02 | 0.01 | 0.233 |
| Monthly Income | 0.06 | 0.02 | 0.013 | 0.02 | 0.02 | 0.181 |
| Health Status | 0.09 | 0.03 | 0.006 | -0.00 | 0.02 | 0.984 |
| Exposure to Vulnerable Portrayals | 0.19 | 0.05 | ＜0.001 | 0.02 | 0.02 | 0.274 |
| Low Involvement | -0.09 | 0.05 | 0.099 |  |  |  |
| High Involvement | 0.17 | 0.07 | 0.008 |  |  |  |
| Exposure×Low Involvement | -0.09 | 0.07 | 0.199 |  |  |  |
| Exposure×Exposure | -0.19 | 0.08 | 0.013 |  |  |  |
| Overall Stereotypes |  |  |  | 0.86 | 0.03 | ＜0.001 |
| *F* | 9.36^***^ | | | 145.97^***^ | | |
| *R*^2^ | 0.16 | | | 0.68 | | |

#### Section 2c. Bootstrap mediating effect results for Studies 1-4

| **Study** | **Conditions of paths** | **Indirect Effect** | | **CI at 95% Level** | |
| --- | --- | --- | --- | --- | --- |
| Study 1 | Low Involvement | 0.085 | | -0.002 | 0.168 |
|  | **Moderate Involvement** | **0.166** | | **0.052** | **0.285** |
|  | High Involvement | 0.003 | | -0.080 | 0.097 |
| Study 2 | Low Involvement | -0.026 | | -0.095 | 0.044 |
|  | **Moderate Involvement** | **0.084** | | **0.011** | **0.164** |
|  | High Involvement | 0.014 | | -0.079 | 0.107 |
|  | Super High Involvement | -0.123 | | -0.291 | 0.037 |
| Study 3a | Low Involvement | -0.030 | | -0.129 | 0.069 |
|  | **Moderate Involvement** | **0.131** | | **0.030** | **0.236** |
| Study 3b | No Involvement | 0.069 | | -0.051 | 0.182 |
|  | Low Involvement | 0.016 | | -0.101 | 0.131 |
|  | **Moderate Involvement** | **0.157** | | **0.050** | **0.297** |
| Study 4 | Vulnerable Group | 0.067 | -0.027 | | 0.173 |
|  | **Vulnerable Group with High Involvement** | **0.133** | **0.023** | | **0.257** |
| **Note**: Bold indicates that 95% CI of the path does not contain 0. | | | | | |

### Section 3: Supplemental Results for Study 2

#### Section 3a. Descriptive statistics and correlation in Study 2

|  | *M* | *SD* | 1 | 2 | 3 | 4 | 5 | 6 | 7 | 8 | 9 | 10 | 11 | 12 |
| --- | --- | --- | --- | --- | --- | --- | --- | --- | --- | --- | --- | --- | --- | --- |
| 1 Portrayals | - | - | - |  |  |  |  |  |  |  |  |  |  |  |
| 2 Enduring Involvement | 2.60 | 0.96 | -0.03 |  |  |  |  |  |  |  |  |  |  |  |
| 3 Sex | 0.38 | 0.49 | -0.02 | -0.09* |  |  |  |  |  |  |  |  |  |  |
| 4 Age | 34.47 | 11.57 | -0.05 | -0.06 | 0.09* |  |  |  |  |  |  |  |  |  |
| 5 Education level | 5.66 | 1.10 | 0.01 | 0.08 | -0.02 | -0.36*** |  |  |  |  |  |  |  |  |
| 6 Income | 3.49 | 1.10 | -0.03 | 0.01 | 0.05 | -0.04 | 0.41*** |  |  |  |  |  |  |  |
| 7 Health Status | 3.78 | 0.83 | -0.01 | -0.29*** | 0.04 | -0.17** | 0.10** | 0.11** |  |  |  |  |  |  |
| 8 Daily Exposure | 2.83 | 0.99 | 0.08* | 0.31*** | -0.05 | -0.20** | 0.19** | 0.07* | -0.06 |  |  |  |  |  |
| 9 Warmth | 3.79 | 0.82 | -0.02 | 0.07 | 0.12** | 0.15*** | -0.08* | -0.05 | 0.09* | 0.00 |  |  |  |  |
| 10 Competence | 4.23 | 0.65 | -0.03 | 0.06 | 0.02 | 0.11** | -0.08* | 0.03 | 0.04 | -0.04 | 0.53*** |  |  |  |
| 12 Morality | 4.18 | 0.70 | -0.03 | 0.16*** | 0.00 | 0.05 | -0.03 | 0.02 | 0.06 | 0.03 | 0.61*** | 0.58*** |  |  |
| 12 Overall Stereotypes | 4.06 | 0.61 | -0.03 | 0.12** | 0.06 | 0.13** | -0.08* | -0.01 | 0.07* | 0.00 | 0.87*** | 0.81*** | 0.86*** |  |
| 13 Trust in Doctors | 3.82 | 0.61 | 0.05 | 0.18*** | 0.03 | -0.06 | 0.00 | 0.14*** | 0.15*** | 0.10** | 0.45*** | 0.42*** | 0.49*** | 0.54*** |
| **Note：****p*＜0.05,***p*＜0.01,****p*＜0.001. Since hospital patients could not quantify their involvement level, this involvement level in this table only included the general public data collected online. | | | | | | | | | | | | | | |

####

#### Section 3b. Post hoc tests of different involvement groups in Study 2

| Dependent variables | *M* | | | | *t* | | | | | |
| --- | --- | --- | --- | --- | --- | --- | --- | --- | --- | --- |
|  | 1 | 2 | 3 | 4 | 1-2 | 1-3 | 1-4 | 2-3 | 2-4 | 3-4 |
| Warmth | 4.14 | 3.79 | 3.77 | 3.64 | 3.33*** | 3.73*** | 5.89*** | 0.10 | 1.72† | 1.82† |
| Competence | 4.40 | 4.26 | 4.21 | 4.16 | 2.05* | 3.53*** | 6.40*** | 1.25 | 3.70*** | 2.59* |
| Morality | 4.47 | 4.29 | 4.18 | 4.01 | 1.69† | 2.47* | 4.16*** | 0.61 | 1.44 | 0.85 |
| Overall Stereotypes | 4.34 | 4.11 | 4.05 | 3.94 | 2.88** | 3.91*** | 6.37*** | 0.74 | 2.70** | 2.11* |
| Trust in Doctors | 3.85 | 3.98 | 3.87 | 3.72 | -1.68† | -0.39 | 2.09* | 1.39 | 3.96*** | 2.72** |
| **Note：**†*p*＜0.1，**p*＜0.05,***p*＜0.01,****p*＜0.001. 1=super high involvement group, 2=high involvement group,3=moderate involvement group,4=low involvement group. | | | | | | | | | | |

#### Section 3c. The moderated mediating models of Study 2

|  | Model 1：Overall Stereotypes | | | Model 2：Trust in Doctors | | |
| --- | --- | --- | --- | --- | --- | --- |
|  | *B* | *se* | *p* | *B* | *se* | *p* |
| Sex | 0.07 | 0.05 | 0.121 | -0.00 | 0.04 | 0.924 |
| Age | 0.00 | 0.00 | 0.433 | -0.01 | 0.00 | ＜0.001 |
| Education Level | -0.01 | 0.02 | 0.564 | -0.05 | 0.02 | 0.019 |
| Income | 0.00 | 0.02 | 0.827 | 0.09 | 0.02 | ＜0.001 |
| Health Status | 0.11 | 0.03 | ＜0.001 | 0.06 | 0.02 | 0.011 |
| Daily Exposure to Vulnerable Portrayals | 0.01 | 0.02 | 0.565 | 0.05 | 0.02 | 0.007 |
| Portrayal | 0.16 | 0.09 | 0.095 | 0.07 | 0.04 | 0.064 |
| Low Involvement | -0.04 | 0.08 | 0.633 |  |  |  |
| High Involvement | 0.17 | 0.10 | 0.093 |  |  |  |
| Super High Involvement | 0.48 | 0.11 | ＜0.001 |  |  |  |
| Portrayal×Low Involvement | -0.20 | 0.12 | 0.076 |  |  |  |
| Portrayal×High Involvement | -0.13 | 0.15 | 0.370 |  |  |  |
| Portrayal×Super High Involvement | -0.39 | 0.14 | 0.006 |  |  |  |
| Overall Stereotypes |  |  |  | 0.54 | 0.03 | ＜0.001 |
| *F* | 5.49^***^ | | | 45.50^***^ | | |
| *R*^2^ | 0.09 | | | 0.34 | | |

### Section 4: Supplemental Results for Study 3a

#### Section 4a. Descriptive statistics and correlation in Study 3a

|  | *M* | *SD* | 1 | 2 | 3 | 4 | 5 | 6 | 7 | 8 | 9 | 10 | 11 | 12 |
| --- | --- | --- | --- | --- | --- | --- | --- | --- | --- | --- | --- | --- | --- | --- |
| 1 Portrayal | - | - |  |  |  |  |  |  |  |  |  |  |  |  |
| 2 Involvement | - | - | 0.03 |  |  |  |  |  |  |  |  |  |  |  |
| 3 Sex | - | - | -0.12* | -0.03 |  |  |  |  |  |  |  |  |  |  |
| 4 Age | 31.03 | 6.33 | 0.02 | -0.03 | 0.15** |  |  |  |  |  |  |  |  |  |
| 5 Education | 5.93 | 0.67 | -0.01 | 0.08 | 0.01 | -0.16** |  |  |  |  |  |  |  |  |
| 6 Income | 3.80 | 1.55 | -0.02 | 0.00 | 0.13** | 0.27*** | 0.30*** |  |  |  |  |  |  |  |
| 7 Health | 4.02 | 0.66 | -0.02 | 0.01 | 0.05 | 0.02 | 0.09 | 0.09 |  |  |  |  |  |  |
| 8 Daily Exposure | 3.34 | 1.02 | 0.06 | -0.07 | 0.05 | 0.03 | 0.07 | 0.10* | 0.11* |  |  |  |  |  |
| 9 Warmth | 3.67 | 0.78 | 0.05 | -0.03 | 0.06 | 0.01 | 0.02 | 0.10* | 0.16** | 0.16** |  |  |  |  |
| 10 Morality | 4.10 | 0.69 | 0.06 | -0.01 | -0.01 | -0.06 | 0.10* | 0.12* | 0.20*** | 0.23*** | 0.60*** |  |  |  |
| 11 Competence | 4.23 | 0.61 | 0.08 | 0.02 | -0.13** | -0.11* | 0.12* | -0.01 | 0.11* | 0.14** | 0.23*** | 0.33*** |  |  |
| 12 Overall Stereotypes | 4.00 | 0.54 | 0.08 | -0.01 | -0.02 | -0.07 | 0.10* | 0.10 | 0.20*** | 0.23*** | 0.83*** | 0.84*** | 0.63*** |  |
| 13 Trust | 3.60 | 0.74 | 0.10* | -0.01 | -0.04 | 0.02 | 0.10* | 0.18*** | 0.21*** | 0.27*** | 0.48*** | 0.48*** | 0.37*** | 0.58*** |

#### Section 4b. Results of two-way ANOVA for Study 3a

| Dependent variables | Main effect of Portrayal | | | | Main effect of involvement | | | | Interaction effect | | |
| --- | --- | --- | --- | --- | --- | --- | --- | --- | --- | --- | --- |
|  | *F* | *p* | η^2^p | *F* | | *p* | η^2^p | *F* | | *p* | η^2^p |
| Warmth | 0.847 | 0.358 | 0.002 | 0.368 | | 0.544 | 0.001 | 4.638 | | 0.023 | 0.011 |
| Competence | 2.133 | 0.145 | 0.005 | 0.055 | | 0.815 | 0.000 | 1.914 | | 0.167 | 0.005 |
| Morality | 1.470 | 0.226 | 0.004 | 0.027 | | 0.870 | 0.000 | 2.209 | | 0.138 | 0.005 |
| Stereotypes | 2.319 | 0.129 | 0.006 | 0.076 | | 0.784 | 0.000 | 4.875 | | 0.028 | 0.012 |
| Trust | 4.255 | 0.040 | 0.011 | 0.078 | | 0.780 | 0.000 | 0.303 | | 0.582 | 0.001 |

#### Section 4c. The moderated mediating models of Study 3a

|  | Model 1：Overall Stereotypes | | | Model 2：Trust | | |
| --- | --- | --- | --- | --- | --- | --- |
|  | *B* | *se* | *p* | *B* | *se* | *p* |
| Sex | -0.02 | 0.05 | 0.671 | -0.06 | 0.06 | 0.327 |
| Age | -0.01 | 0.00 | 0.091 | 0.00 | 0.01 | 0.597 |
| Education Level | 0.02 | 0.04 | 0.596 | 0.01 | 0.05 | 0.889 |
| Income | 0.03 | 0.02 | 0.171 | 0.05 | 0.02 | 0.024 |
| Health Status | 0.14 | 0.04 | ＜0.001 | 0.11 | 0.05 | 0.021 |
| Daily Exposure | 0.11 | 0.03 | ＜0.001 | 0.09 | 0.03 | 0.003 |
| Enduring Involvement | 0.04 | 0.04 | 0.371 | 0.08 | 0.05 | 0.098 |
| Portrayal | -0.04 | 0.07 | 0.573 | 0.08 | 0.06 | 0.189 |
| Involvement | -0.12 | 0.07 | 0.097 |  |  |  |
| Portrayal×Involvement | 0.23 | 0.10 | 0.027 |  |  |  |
| Overall Stereotypes |  |  |  | 0.71 | 0.06 | ＜0.001 |
| *F* | 5.18^***^ | | | 27.12^***^ | | |
| *R*^2^ | 0.12 | | | 0.38 | | |

### Section 5: Supplemental Results for Study 3b

#### Section 5a. Descriptive statistics and correlation in Study 3b

|  | *M* | *SD* | 1 | 2 | 3 | 4 | 5 | 6 | 7 | 8 | 9 | 10 | 11 | 12 | 13 | 14 | 15 | 16 |
| --- | --- | --- | --- | --- | --- | --- | --- | --- | --- | --- | --- | --- | --- | --- | --- | --- | --- | --- |
| 1 Portrayal | - | - |  |  |  |  |  |  |  |  |  |  |  |  |  |  |  |  |
| 2 Involvement | - | - | 0.04 |  |  |  |  |  |  |  |  |  |  |  |  |  |  |  |
| 3 Sex | - | - | 0.03 | -0.06 |  |  |  |  |  |  |  |  |  |  |  |  |  |  |
| 4 Age | 22.52 | 2.45 | -0.02 | 0.04 | 0.24^**^ |  |  |  |  |  |  |  |  |  |  |  |  |  |
| 5 Education | 6.54 | 0.83 | -0.01 | 0.09 | 0.10 | 0.62^***^ |  |  |  |  |  |  |  |  |  |  |  |  |
| 6 Income | 1.62 | 0.79 | 0.17^*^ | 0.06 | 0.14 | 0.39^***^ | 0.22^**^ |  |  |  |  |  |  |  |  |  |  |  |
| 7 Health | 4.11 | 0.65 | 0.00 | -0.09 | 0.10 | 0.14 | 0.13 | 0.06 |  |  |  |  |  |  |  |  |  |  |
| 8 Daily Exposure | 2.66 | 0.83 | 0.20^*^ | -0.12 | 0.08 | 0.19^*^ | 0.12 | 0.22^**^ | -0.07 |  |  |  |  |  |  |  |  |  |
| 9 T1 Warmth | 3.44 | 0.69 | 0.06 | -0.08 | 0.25^**^ | 0.06 | -0.05 | -0.01 | 0.06 | 0.15 |  |  |  |  |  |  |  |  |
| 10 T1 Morality | 4.02 | 0.68 | 0.14 | -0.18^*^ | -0.02 | -0.04 | -0.08 | 0.00 | -0.03 | 0.10 | 0.49^***^ |  |  |  |  |  |  |  |
| 11 T1 Competence | 4.14 | 0.73 | 0.08 | -0.07 | -0.05 | -0.10 | -0.13 | 0.09 | -0.07 | 0.14 | 0.40^***^ | 0.62^***^ |  |  |  |  |  |  |
| 12 T1 Stereotypes | 3.87 | 0.57 | 0.11 | -0.13 | 0.07 | -0.04 | -0.11 | 0.04 | -0.02 | 0.16^*^ | 0.77^***^ | 0.86^***^ | 0.83^***^ |  |  |  |  |  |
| 13 T2 Warmth | 3.57 | 0.67 | 0.19^*^ | -0.15 | 0.29^***^ | 0.02 | -0.13 | 0.05 | 0.14 | 0.28^***^ | 0.65^***^ | 0.33^***^ | 0.21^**^ | 0.48^***^ |  |  |  |  |
| 14 T2 Morality | 4.11 | 0.62 | 0.26^**^ | -0.21^**^ | 0.05 | 0.01 | -0.04 | 0.03 | 0.05 | 0.16^*^ | 0.33^**^ | 0.70^**^ | 0.35^**^ | 0.56^**^ | 0.50^***^ |  |  |  |
| 15 T2 Competence | 4.14 | 0.65 | 0.17^*^ | -0.18^*^ | 0.01 | -0.07 | -0.10 | 0.04 | -0.02 | 0.24^**^ | 0.24^**^ | 0.39^***^ | 0.68^***^ | 0.54^***^ | 0.40^***^ | 0.53^***^ |  |  |
| 16 T2 Stereotypes | 3.94 | 0.52 | 0.25^**^ | -0.23^**^ | 0.14 | -0.02 | -0.11 | 0.05 | 0.07 | 0.28^***^ | 0.51^***^ | 0.58^***^ | 0.51^***^ | 0.65^***^ | 0.79^***^ | 0.83^***^ | 0.80^***^ |  |
| 17 Trust | 3.16 | 0.65 | 0.17^*^ | -0.17^*^ | 0.17^*^ | -0.05 | -0.05 | 0.10 | 0.10 | 0.19^*^ | 0.20^*^ | 0.20^*^ | 0.26^**^ | 0.27^**^ | 0.30^***^ | 0.28^***^ | 0.33^***^ | 0.38^***^ |

#### Section 5b. The ANOVA results of Study 3b

Utilizing the level of involvement as the independent variable, a variance analysis was conducted on the three dimensions and the overall evaluations at Time 1. The results reveal that the main effect of involvement is only marginally significant in the dimension of moral evaluations, *F*(2, 157) = 2.76, *p* = 0.066. Post hoc comparisons indicated that, in the high-involvement group, the moral evaluations (*M* = 3.86) were significantly worse than those in the no-involvement group (*M* = 4.15, *t*(157) = -2.24, *p* = 0.026, Cohen’s *d* = -0.43) and marginally worse than those in the low-involvement group (*M* = 4.08, *t*(157) = -1.70, *p* = 0.090, Cohen’s *d* = -0.33). This suggests that this paradigm of manipulation of imaginative involvement might exert a certain degree of negative influence on the moral evaluations of doctors.

Employing both the portrayal and the level of involvement as independent variables, a variance analysis was conducted on the three dimensions and the overall evaluations at Time 2. The findings indicate a significant main effect of vulnerable portrayal on overall evaluations and its three dimensions (overall evaluations: *F*(1, 154) = 11.69, *p* < 0.001; warmth: *F*(1, 154) = 5.87, *p* = 0.017; competence: *F* (1, 154) = 4.76, *p* = 0.031; morality: *F* (1, 154) = 12.37, *p* < 0.001). The vulnerable group exhibited significantly better evaluations and three dimensions than the control group.

Although the interaction effect between vulnerable portrayal and the level of situational involvement was not significant for overall medical evaluations or its three dimensions (*F*s < 2.10, *p*s > 0.12), further simple effect tests revealed (see Figure 1) that after the high-involvement manipulation, the vulnerable group had significantly higher scores for overall evaluations and its three dimensions than the control group (overall evaluations: *t*(154) = 3.48, *p* < 0.001, Cohen’s *d* = 0.93; warmth: *t*(154) = 2.69, *p* = 0.008, Cohen’s *d* = 0.72; competence: *t*(154) = 2.43, *p* = 0.016, Cohen’s *d* = 0.65; morality: *t*(154) = 3.11, *p* = 0.002, Cohen’s *d* = 0.83), supporting H7a to 7d. Conversely, between the low-involvement vulnerable group and the control group, there was no significant disparity in medical evaluations or trust, supporting H6a to 6d. Moreover, it is noteworthy that without the involvement manipulation, the vulnerable group’s moral evaluations of doctors were significantly better than those of the control group, *t*(154) = 2.38, *p* = 0.019, Cohen’s *d* = 0.67, and the overall evaluations were marginally significantly better for the vulnerable group than for the control group, *t*(154) = 1.96, *p* = 0.052, Cohen’s *d* = 0.55.

**Figure 1 Medical Evaluation Scores for Each Group in Study**

Two-way analysis of variance was conducted with trust as the dependent variable. The results revealed a significant main effect of vulnerable portrayal, *F*(1, 154) = 4.88, *p* = 0.029. Participants exposed to the vulnerable medical professional portrayal exhibited greater trust in doctors (*M* = 3.27) than those in the control group (*M* = 3.06). Moreover, the main effect of involvement was also significant, *F*(2, 154) = 3.68, *p* = 0.028. Trust in doctors was significantly lower in the high-involvement group (*M* = 2.98) than in the low-involvement group (*M* = 3.27, *t*(157) = -2.38, *p* = 0.019, Cohen’s *d* = -0.46) or in the no-involvement group (*M* = 3.26, *t*(157) = -2.29, *p* = 0.023, Cohen’s *d* = -0.44). Although the interaction effect was not significant, *F*(2, 154) = 0.83, *p* = 0.44, further simple effect analysis still indicated that after the moderate-involvement manipulation, the trust of the vulnerable group was significantly higher than that of the control group, *t*(154) = 2.37, *p* = 0.019, Cohen’s *d* = 0.64.

#### Section 5c. The moderated mediating models of Study 3b

|  | Model 1：Overall Stereotypes | | | Model 2：Trust | | |
| --- | --- | --- | --- | --- | --- | --- |
|  | *B* | *se* | *p* | *B* | *se* | *p* |
| Sex | 0.12 | 0.09 | 0.169 | 0.18 | 0.11 | 0.109 |
| Age | 0.01 | 0.02 | 0.759 | -0.04 | 0.03 | 0.140 |
| Education Level | -0.09 | 0.06 | 0.144 | 0.02 | 0.07 | 0.828 |
| Income | -0.03 | 0.06 | 0.629 | 0.06 | 0.07 | 0.366 |
| Health Status | 0.05 | 0.06 | 0.456 | 0.07 | 0.08 | 0.355 |
| Daily Exposure | 0.15 | 0.05 | 0.003 | 0.09 | 0.06 | 0.177 |
| Enduring Involvement | -0.03 | 0.05 | 0.550 | -0.04 | 0.07 | 0.541 |
| Portrayal | 0.18 | 0.14 | 0.194 | 0.06 | 0.10 | 0.560 |
| Low Involvement | -0.11 | 0.13 | 0.424 |  |  |  |
| Moderate Involvement | -0.35 | 0.13 | 0.009 |  |  |  |
| Portrayal×Low Involvement | -0.14 | 0.19 | 0.465 |  |  |  |
| Portrayal×Moderate Involvement | 0.23 | 0.19 | 0.218 |  |  |  |
| Overall Stereotypes |  |  |  | 0.38 | 0.10 | ＜0.001 |
| *F* | 3.46^***^ | | | 3.90^***^ | | |
| *R*^2^ | 0.22 | | | 0.19 | | |

### A regression model was employed with Model 7 selected and the involvement group as the moderating variable (no involvement coded as 00). Although no significant moderation effect was observed, further simple slope analysis revealed (see Figure 2) that when no situational involvement manipulation was present, the effect of vulnerable portrayal on evaluations was not significant (*simple slope* = 0.18, *se* = 0.14, *p* = 0.19). Similarly, under low situational involvement, the predictive effect of vulnerable portrayal on medical evaluations was also not significant (*simple slope* = 0.04, *se* = 0.13, *p* = 0.76). Only in the case of high situational involvement did vulnerable portrayal have a positive impact on evaluations (*simple slope* = 0.42, *se* = 0.13, *p* = 0.002), which further positively influenced trust in doctors (*b* = 0.38, p< 0.001); the indirect effect was 0.16, 95% CI [0.05,0.30], supporting H4 and H5.

**Figure 2 Simple Slope Plot of Study 3b**

### Section 6: Supplemental Results for Study 4

#### Section 6a. Descriptive statistics and correlation in Study 4

|  | *M* | *SD* | 1 | 2 | 3 | 4 | 5 | 6 | 7 | 8 | 9 | 10 | 11 |
| --- | --- | --- | --- | --- | --- | --- | --- | --- | --- | --- | --- | --- | --- |
| 1 Sex | - | - |  |  |  |  |  |  |  |  |  |  |  |
| 2 Age | 30.04 | 7.95 | 0.24*** |  |  |  |  |  |  |  |  |  |  |
| 3 Education | 6.03 | 0.66 | -0.06 | 0.01 |  |  |  |  |  |  |  |  |  |
| 4 Income | 3.55 | 1.01 | 0.11* | 0.21*** | 0.17*** |  |  |  |  |  |  |  |  |
| 5 Health | 4.18 | 0.60 | 0.03 | 0.05 | 0.12* | 0.16** |  |  |  |  |  |  |  |
| 6 Daily Exposure | 2.95 | 0.99 | -0.05 | -0.13** | -0.14** | -0.05 | -0.28*** |  |  |  |  |  |  |
| 7 Enduring Involvement | 2.17 | 0.62 | -0.06 | 0.01 | 0.02 | 0.14** | -0.22*** | 0.20*** |  |  |  |  |  |
| 8 Warmth | 3.73 | 0.68 | 0.13** | 0.11* | 0.06 | 00.05 | 0.31*** | -0.06 | -0.03 |  |  |  |  |
| 9 Competence | 4.39 | 0.55 | 0.07 | 0.06 | 0.03 | 00.09 | 0.30*** | -0.06 | -0.07 | 0.34*** |  |  |  |
| 10 Morality | 4.19 | 0.58 | 0.13** | 0.07 | 0.11* | 0.13** | 0.32*** | -0.14** | -0.06 | 0.58*** | 0.44*** |  |  |
| 11 Overall Stereotypes | 4.10 | 0.48 | 0.14** | 0.10* | 0.08 | 0.11* | 0.39*** | -0.11* | -00.06 | 0.83*** | 0.72*** | 0.84*** |  |
| 12 Trust | 4.07 | 0.60 | 0.11* | 0.11* | 0.12* | 0.14** | 0.40*** | -0.25*** | -0.09* | 0.68*** | 0.48*** | 0.74*** | 0.80*** |
| **Note：****p*＜0.05,***p*＜0.01,****p*＜0.001. | | | | | | | | | | | | | |

#### Section 6b. Results of one-way ANOVA for Study 4

| Dependent variables | *F* | *p* | η^2^p |
| --- | --- | --- | --- |
| Warmth | 4.501 | 0.012 | 0.020 |
| Competence | 1.555 | 0.212 | 0.007 |
| Morality | 3.895 | 0.021 | 0.018 |
| Stereotypes | 4.978 | 0.007 | 0.022 |
| Trust | 2.194 | 0.113 | 0.010 |

#### Section 6c. The moderated mediating models of Study 4

|  | Model 1：Overall Stereotypes | | | | Model 2：Trust | | |
| --- | --- | --- | --- | --- | --- | --- | --- |
|  | *B* | *se* | *p* | *B* | | *se* | *p* |
| Sex | 0.11 | 0.04 | 0.017 | -0.01 | | 0.04 | 0.754 |
| Age | 0.00 | 0.00 | 0.173 | -0.00 | | 0.00 | 0.900 |
| Education Level | 0.03 | 0.03 | 0.351 | 0.02 | | 0.03 | 0.561 |
| Income | 0.01 | 0.02 | 0.786 | 0.03 | | 0.02 | 0.098 |
| Health Status | 0.30 | 0.04 | ＜0.001 | 0.05 | | 0.03 | 0.105 |
| Daily Exposure | 0.00 | 0.02 | 0.991 | -0.09 | | 0.02 | ＜0.001 |
| Enduring Involvement | 0.02 | 0.04 | 0.548 | -0.01 | | 0.03 | 0.600 |
| Vulnerable Portrayals | 0.07 | 0.05 | 0.169 | -0.08 | | 0.04 | 0.065 |
| Vulnerable Portrayals with High Involvement | 0.14 | 0.05 | 0.006 | -0.02 | | 0.04 | 0.603 |
| Overall Stereotypes |  |  |  | 0.93 | | 0.04 | ＜0.001 |
| *F* | 10.87^***^ | | | | 86.03^***^ | | |
| *R*^2^ | 0.19 | | | | 0.67 | | |

### Section 7: Confirmatory Factor Analysis of Studies 1-4

#### Section 7a. Common Method Bias and CFA of Study 1

To address the potential influence of common method bias stemming from self-reported data, a Harman single-factor test was employed. The Kaiser-Meyer-Olkin (KMO) value was found to be 0.92, and Bartlett's test of sphericity showed a significance level of P < 0.001, indicating the suitability of conducting factor analysis. An exploratory factor analysis was conducted on all items without rotation, revealing that the variance explained by the first common factor was 31.83%, falling below the critical threshold of 40%. As a result, it can be concluded that significant common method bias is unlikely to exist in the data for this study.

Moreover, using Amos 21.0, confirmatory factor analyses were conducted based on the sample data to construct stereotype content models (SCMs) with single-factor, two-factor, and three-factor solutions. The maximum likelihood estimation method was employed to evaluate the models. The outcomes are presented in Table 7-1. The fit indices for the single-factor model and the two-factor model, which combined moral dimension items into the warmth dimension, exhibited poorer performance. Conversely, the three-factor model displayed favorable fit indices, supporting the hypothesis that the stereotype content model encompasses three distinct dimensions. The standardized factor loadings for the three-factor model all exceeded 0.7, as illustrated in Figure 7-1.

Table 1 SCM confirmatory factor analysis model comparison fitting index（*N*=492）

|  | χ^2^/*df* | SRMR | RMSEA | CFI | TLI | AIC | BIC |
| --- | --- | --- | --- | --- | --- | --- | --- |
| One-Factor Model | 12.487 | 0.0593 | 0.152 | 0.907 | 0.845 | 136.384 | 186.888 |
| Two-Factor Model | 9.04 | 0.0476 | 0.127 | 0.942 | 0.891 | 98.316 | 153.028 |
| Three-Factor Model | 5.557 | 0.0303 | 0.096 | 0.975 | 0.939 | 63.34 | 126.469 |


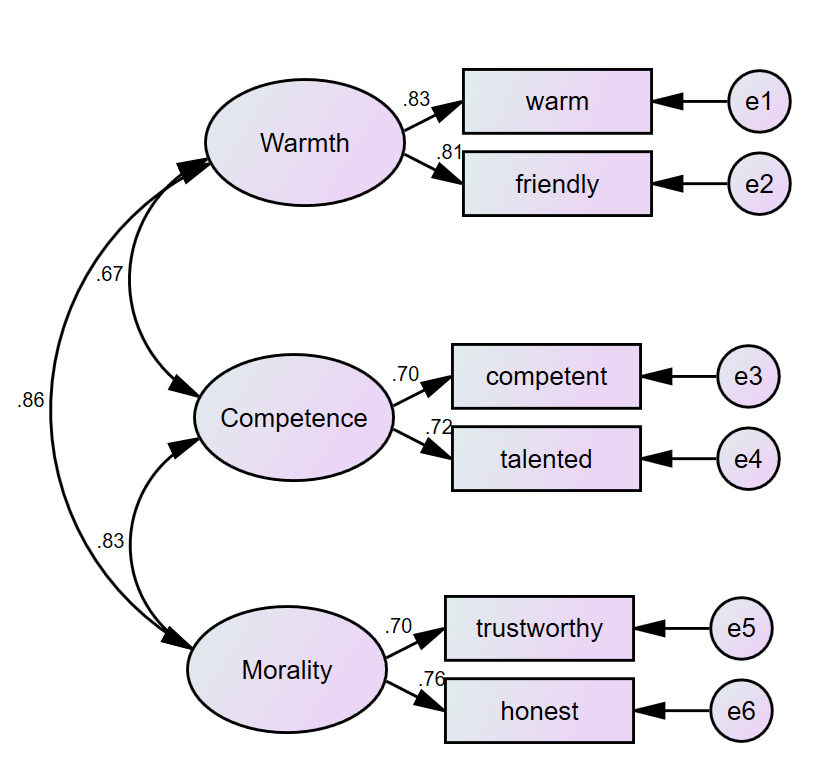
Figure 7-1 Fitting effect drawing of confirmatory factor analysis for three-factor SCM

#### Section 7b. CFA of Studies 2-4

Similar to Study 1, Confirmatory Factor Analysis (CFA) was conducted in AMOS 21.0 based on the sample data from Studies 2-4 to construct stereotype content models (SCMs) with 1 to 3 factors.

In Study 2, The results indicated that the fit indices for the single-factor model (χ2/df = 15.69, CFI = 0.921, TLI = 0.815, RMSEA = 0.144) and the two-factor model (χ2/df = 13.13, CFI = 0.942, TLI = 0.847, RMSEA = 0.131) were unsatisfactory. In contrast, the three-factor model exhibited excellent fit indices, withχ2/df = 2.91, CFI = 0.993, TLI = 0.976, and RMSEA = 0.052. These findings once again provided support for the hypothesis that warmth and morality are distinct dimensions and not identical.

In Study 3a, the results demonstrated that the fit indices for the single-factor model (χ2/df = 10.38, CFI = 0.863, TLI = 0.771, RMSEA = 0.152, SRMR = 0.079) and the two-factor model (χ2/df = 5.88, CFI = 0.936, TLI = 0.881, RMSEA = 0.110, SRMR = 0.046) were not satisfactory. Conversely, the three-factor model exhibited excellent fit indices, with χ2/df = 2.97, CFI = 0.981, TLI = 0.952, RMSEA = 0.070, and SRMR = 0.030.

In Study 3b, compared to the single-factor model (T1: χ2/df = 9.12, CFI = 0.802, TLI = 0.670, RMSEA = 0.226, SRMR = 0.091; T2: χ2/df = 10.59, CFI = 0.763, TLI = 0.605, RMSEA = 0.246, SRMR = 0.097) and the two-factor model (T1: χ2/df = 7.60, CFI = 0.857, TLI = 0.732, RMSEA = 0.204, SRMR = 0.079; T2: χ2/df = 8.40, CFI = 0.837, TLI = 0.695, RMSEA = 0.216, SRMR = 0.079), the three-factor model consistently yielded better fit indices (T1: χ2/df = 3.27, CFI = 0.963, TLI = 0.908, RMSEA = 0.119, SRMR = 0.042; T2: χ2/df = 3.524, CFI = 0.958, TLI = 0.896, RMSEA = 0.126, SRMR = 0.046).

The results of Study 4 similarly indicate that the three-factor model yielded the best fit indices, with χ2/df = 2.81, CFI = 0.981, TLI = 0.953, RMSEA = 0.064, and SRMR = 0.026. All the results of 4 studies reinforces the hypothesis that the stereotype content model encompasses three dimensions.
